# Immune inflammatory regulation in Anti-NMDAR encephalitis: insights from transcriptome analysis

**DOI:** 10.3389/fneur.2025.1568274

**Published:** 2025-05-09

**Authors:** Shan Qiao, Jia Wang, Shan-chao Zhang, Ai-hua Wang, Hai-yun Li, Tao Xin

**Affiliations:** ^1^Department of Neurology, The First Affiliated Hospital of Shandong First Medical University & Shandong Provincial Qianfoshan Hospital, Jinan, China; ^2^Post-Doctoral Scientific Research Station, Shandong University of Traditional Chinese Medicine, Jinan, Shandong, China; ^3^Human Resource Department, The First Affiliated Hospital of Shandong First Medical University & Shandong Provincial Qianfoshan Hospital, Jinan, China; ^4^Department of Geriatric Medicine, Qilu Hospital of Shandong University, Jinan, China; ^5^Department of Neurosurgery, The First Affiliated Hospital of Shandong First Medical University & Shandong Provincial Qianfoshan Hospital, Jinan, China; ^6^Medical Science and Technology Innovation Center, Shandong First Medical University and Shandong Academy of Medical Sciences, Jinan, China

**Keywords:** anti-N-Methyl-D-aspartate receptor encephalitis, diagnostic marker, transcriptomics, immune cell infiltration, neutrophils

## Abstract

**Background:**

Anti-N-methyl-D-aspartate receptor (NMDAR) encephalitis is a critical neurological disorder mediated by autoimmune mechanisms, Previous literature suggests that immune inflammatory responses may be involved in the progression of anti NMDAR encephalitis, but its molecular regulatory mechanisms still remain uncertain. We aimed to identify transcriptome-wide landscape of mRNAs and explore the potential pathogenesis for anti-NMDAR encephalitis.

**Methods:**

Peripheral blood mononuclear cells were obtained from six patients with anti-NMDAR encephalitis and six controls for RNA extraction and library creation. The Illumina HiSeq platform was used to do transcriptome sequencing. We utilized R software to identify differentially expressed genes (DEGs) and performed a functional enrichment analysis. Furthermore, random forest (RF) and support vector machine-recursive feature elimination (SVM-RFE) were employed to screen for and identify anti-NMDAR encephalitis diagnostic signatures. To verify the findings, we employed quantitative real-time polymerase chain reaction. Receiver operating characteristic curves were utilized to assess the diagnostic values. We evaluated the inflammatory state of anti-NMDAR encephalitis using cell-type identification by computing the relative subsets of RNA transcripts (CIBERSORT) and investigated the relationship between diagnostic biomarkers and immune cell subsets.

**Results:**

899 DEGs were identified (568 upregulated and 331 downregulated), of which 78 were immune-related genes. The DEGs were found to be considerably enriched in immunological inflammation-related pathways, according to the functional enrichment analysis. Insulin-like factor 3 [area under the curve (AUC) = 0.917] and tumor protein translationally controlled regulator 1 (AUC = 0.944) were considered potential diagnostic indicator candidates of anti-NMDAR encephalitis, with statistically significant variations in expression. An immune cell analysis of immune cell proportions suggests that monocytes, CD8^+^ T cells, and T regulatory cells may all be involved in the development of anti-NMDAR encephalitis.

**Conclusions:**

Transcriptome analysis reveals significant activation of peripheral immune-inflammatory pathways in anti-NMDAR encephalitis. INSL3 and TPT1 may serve as potential auxiliary diagnostic biomarkers, while monocyte, CD8+ T cell, and Treg infiltration likely synergistically drive disease progression.

## 1 Introduction

Anti-N-methyl-D-aspartate receptor (NMDAR) encephalitis is a serious neurological illness caused by autoimmune processes. It is the most common type of autoimmune encephalitis, and it is marked by behavioral abnormalities, psychosis, memory impairment, seizures, mobility difficulties, autonomic dysfunction, and even coma (1, 2). Immunotherapy is an effective treatment for this condition. However, relapse still occurs in ~20–25% of patients (3, 4). Finding anti-NMDAR antibodies of the immunoglobin G class in the serum or/and cerebrospinal fluid (CSF) is necessary for a conclusive diagnosis. Considerable efforts have been made since its discovery in 2007 to explore the pathogenesis and develop biomarkers to better understand the immunological processes. Previous studies have suggested that tumors (mainly ovarian teratomas) or herpes infections may be potential triggering factors in anti NMDAR encephalitis, and pathogenic antibody mediated immune inflammatory responses may be involved in the progression of the disease. However, understanding of the etiology and pathogenesis of anti-NMDAR encephalitis is still limited (3, 5). Therefore, continued research of its pathogenesis is critical to understanding the disease causation and prognosis.

Transcriptomics is the study of transcripts of specific cells, tissues, and organs at specific growth and developmental stages or under certain physiological conditions (6, 7). RNA is transcribed by all tissues or organ cells in the body, including protein-coding messenger RNA (mRNA) and non-coding RNA (ribosomal RNA, transfer RNA, and long noncoding RNA). Transcriptomics is significant for understanding the mechanisms of gene expression and disease development (8). The use of next-generation sequencing-based RNA sequencing (RNA-seq) technology in studies on the causes of disease and biomarkers has increased recently (8–10). The host genes of dysregulated circular RNAs (circRNAs) are mostly involved in receptor internalization, according to research on the circRNA profiles of juvenile patients with anti-NMDAR encephalitis (11). Moreover, Liu et al. (12) investigated the clinical use of serum exosomal microRNAs for differentiating between viral encephalitis (VE) and anti-NMDAR encephalitis. They discovered that serum C3 in conjunction with serum exosomal miR-140-5p may be a useful diagnostic tool for distinguishing between VE and anti-NMDAR encephalitis. All of the preceding studies imply that the transcriptome is important in the pathogenesis of anti-NMDAR encephalitis; nevertheless, research on the pathophysiology of this disease's transcriptome is limited.

Thus, the current work employed RNA-seq technology to explore the transcriptome-wide landscape of mRNAs to explore the transcriptional pathophysiology and possible treatment targets of the illness.

## 2 Methods

### 2.1 Study design

Included were nine patients diagnosed between January 2021 and June 2022 for anti-NMDAR encephalitis and nine age- and sex-matched control subjects from The First Affiliated Hospital of Shandong First Medical University and Qilu Hospital of Shandong University. The diagnosis of anti-NMDAR encephalitis was made using Graus et al.'s (13) criteria. The inclusion criteria were as follows: (1) at least one clinical symptom, such as seizures, mental disorders, impaired mobility, cognitive dysfunction, or a lower state of consciousness, (2) anti-NMDAR antibody positivity in serum and CSF and (3) excluded those with coexisting autoimmune diseases or infectious encephalitis. Controls were age- and sex-matched healthy volunteers with no history of autoimmune diseases or recent infections. The mean age of patients was 27.9 ± 5.9 years vs. 30.7 ± 6.3 years for controls (*p* = 0.347). Loss to follow-up or missing data were excluded criteria. Prior to immunotherapy, all blood samples were taken from patients who were still experiencing symptoms. The control group's members were registered at health screening centers. Before being used for additional testing, all blood samples were quickly frozen in liquid nitrogen and stored at 80°C. Six pairs of samples were used for transcriptome sequencing, and another three pairs of samples were used for the validation set. All participants received abdominal and pelvic ultrasonography and computed tomography screening, and no participant had a teratoma.

As directed by the manufacturer (Euroimmun, Germany), autoantibodies against NMDAR, LGI1, contactin-associated protein-like 2, GABAB receptor, AMPA1, and AMPA2 were assessed using indirect immunofluorescence in both serum and CSF.

### 2.2 Total RNA extraction, library preparation and transcriptome sequencing

Peripheral blood mononuclear cells (PBMCs) were treated with the Qiagen RNeasy Plus Small Kit (Qiagen) in order to extract total RNA from them. Utilizing spectra produced at 260 and 280 nm optical densities using the NanoDrop 2000 (Thermo Fisher Scientific, Waltham, MA, USA), the quality and quantity of RNA were evaluated. Total RNA was used to prepare RNA samples at 2 g each sample. Following the manufacturer's instructions, sequencing libraries were created using the NEBNext^®^ Ultra^TM^ II RNA Library Prep Kit for Illumina. After qualifying, the libraries were pooled by effective concentration (higher than that of 2nM) and machine data need and sequenced using the Illumina NovaSeq 6000. Image data from a high-throughput sequencer was converted to sequence data using CASAVA base recognition. Pair-end clean reads were aligned to the reference genome index produced by Hisat2 (v2.0.5).

### 2.3 Functional correlation analysis

To identify differentially expressed genes, this work used the Limma R package (http://www.bioconductor.org/packages/release/bioc/html/limma.html); additionally, a volcano plot was produced to illustrate DEGs. DEGs with a *p*-value < 0.05 and |fold change (FC)| >1.2 were considered statistically significant. The Benjamini–Hochberg (BH) method was used to correct the *p*-value, and the adjusted *p*-value (*p*. Adjust) is the *p*-value corrected by BH. To get immunity-related genes, the ImmPort database (https://immport.niaid.nih.gov) was used. The “clusterProfiler” software was used for gene ontology (GO) enrichment (14) and Kyoto Encyclopedia of Genes and Genomes (KEGG) pathway enrichment investigations (15) for biological process and pathway enrichment analysis.

The GO enrichment comprises biological processes, cellular components and molecular functions. For each enriched items, *p* value, enrichment score, Fold and ratio were calculated. The *p* < 0.05 was set as the cutoff criterion for significant enrichment. Using the “single-sample gene set enrichment analysis” (ssGSEA) function from the “GSVA” R package (version 4.1.0) (16), we calculated immune-related scores for every sample and examined the relationship between the scores to obtain a visual representation of the significantly enriched functional pathways' gene expression levels. The statistical results of *p*-value were multiply corrected with Benjamini & Hochberg methods, and the adjusted *p* value of FDR (false discovery rate) was provided.

### 2.4 Diagnostic markers screening and verification

Random forest (RF) (17, 18) and support vector machine-recursive feature elimination (SVM-RFE^)^ (19) were employed to identify novel and critical biomarkers of anti-NMDAR encephalitis. For this investigation, the RF algorithm was implemented using the R package “randomForest,” while the RFE function within the caret package, coupled with 5-fold cross-validation, facilitated the selection of highlighted genes. Furthermore, 5-fold cross-validation and the R package e1071 were utilized in the construction of the SVM classifier. We next chose overlapped genes from the two previously described classification models for further investigation. The researchers used receiver operating characteristic (ROC) curve analysis (20) to look for new and important biomarkers of anti-NMDAR encephalitis. ROC curves (pROC software, version 1.17.0.1) were applied to analyze the biomarkers, and the predictive power of the algorithms was tested by calculating the area under the curve (AUC). A two-sided *p* < 0.05 was utilized to indicate statistical significance.

### 2.5 Quantitative real-time polymerase chain reaction (RT-qPCR) analysis

On an ABI PRISM 7900 Sequence Detection System, RT-qPCR was performed using Green Premix Ex Taq II (TaKaRa Bio) (Applied Biosystems). Forty cycles of 15 s at 95°C and 1 min at 60°C were conducted for the PCR. Furthermore, the 2^−ΔΔCt^ method was employed to calculate the data, with GAPDH acting as an endogenous control. The primers utilized in this work are listed in Supplementary Table 1.

### 2.6 Assessment and correlation analysis of immune cells associated with infiltration

The CIBERSORT algorithm (21), a deconvolution algorithm that uses standard gene expression values (a signature with 547 genes) as a minimal representation for each cell type to calculate cell type proportions alongside mixed cell types using support vector regression, quantified the proportions of 22 immune cell types in samples. Cell types with *p* < 0.05 were deemed significant. Additionally, “ggplot2” was used for immune cell infiltration matrix PCA cluster analysis. A correlation heatmap was created using “corrplot” software to show the relationship between 22 kinds of immune cell subsets. The “ggstatsplot” and “ggplot2” packages were used to examine the Spearman correlation between diagnostic indicators and immune/inflammatory cells and display the results.

### 2.7 Statistical analysis

Software such as SPSS v.26.0 or GraphPad Prism 8.0 were used for statistical analysis. The mean ± standard deviation (SD) represents the continuous variable data. The Fisher's exact test was used to evaluate the differences between the experimental groups. However, Spearman's correlation coefficients were used to assess between-variable correlations. Additionally, the Wilcoxon signed rank test was employed to examine the variation in immune cell ratings between various groups. At *p* < 0.05, statistical significance was determined (two-sided).

## 3 Result

### 3.1 Identification of DEGs between anti-NMDAR encephalitis patients and controls

**Figure 1:**
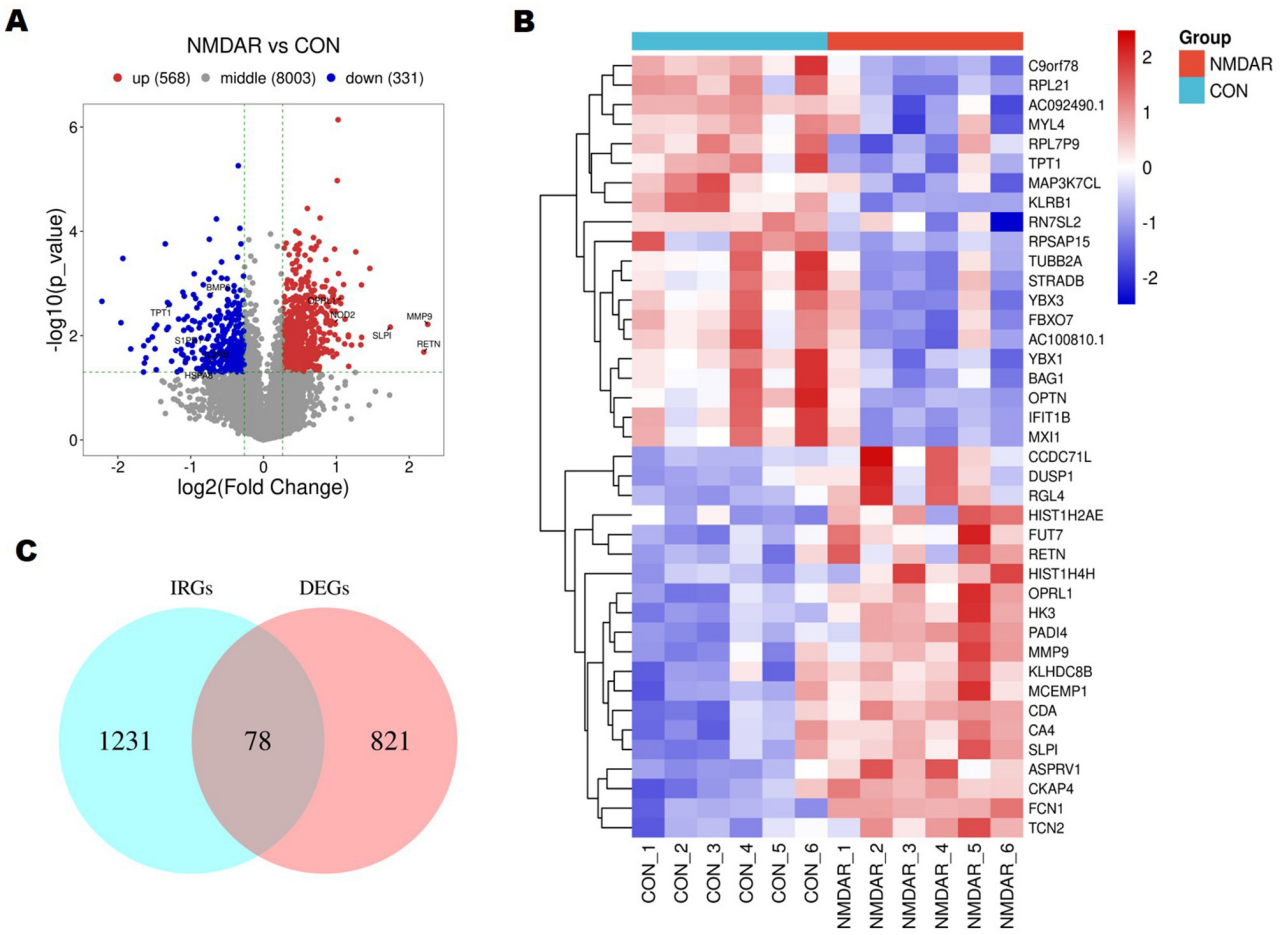
Differentially expressed genes (DEGs) between those suffering from anti-N-methyl-D-aspartate receptor (NMDAR) encephalitis and controls were identified. **(A)** DEGs' volcanic plan. Blue denotes low robust DEG expression, and orange denotes high. The DEGs have marked versions of immune-related genes (IRGs). In all, 899 DEGs, including 568 upregulated and 331 downregulated genes, were discovered. **(B)** Heatmap showing the top 20 DEGs that distinguish patients with anti-NMDAR encephalitis from controls. **(C)** Venn diagram based on the ImmPort database that displays overlapping IRGs in the DEGs. A total of 78 overlapping IRGs in the DEGs were identified.

The DEGs between anti-NMDAR encephalitis individuals and controls were determined. In all, 899 DEGs, including 568 upregulated and 331 downregulated genes, were discovered (Figure 1A, Supplementary Figure 1, and Supplementary Table 2). In addition, the top 20 FC values for DEGs were visualized using a heat map. The top five upregulated DEGs were matrix metallopeptidase 9 (*MMP9*), resistin, secretory leukocyte peptidase inhibitor, peptidyl arginine deiminase 4, and ral guanine nucleotide dissociation stimulator like 4, and the top five downregulated DEGs were *AC092490.1*, ribosomal protein SA pseudogene 15, chromosome 9 open reading frame 78, tubulin beta 2A class IIa, and STE20 related adaptor beta (Figure 1B).

Evidence suggests that immunoregulatory factors mediate the development of anti-NMDAR encephalitis (3, 5). Therefore, we focused on immune-related DEGs to explore the potential pathogenesis of anti-NMDAR encephalitis. By overlapping the 1231 immune-related genes (IRGs) obtained from the ImmPort database, we obtained a total of 78 overlapping IRGs in the DEGs (Figure 1C), including but not limited to *IL2RB*, high mobility group box 1 (*HMGB1*), *MMP9*, S100 calcium binding protein A6, colony stimulating factor 3 receptor (*CSF3R*), interleukin 17A receptor, and tumor necrosis factor receptor superfamily 1A (Supplementary Table 3).

### 3.2 Functional enrichment analyses

**Figure 2:**
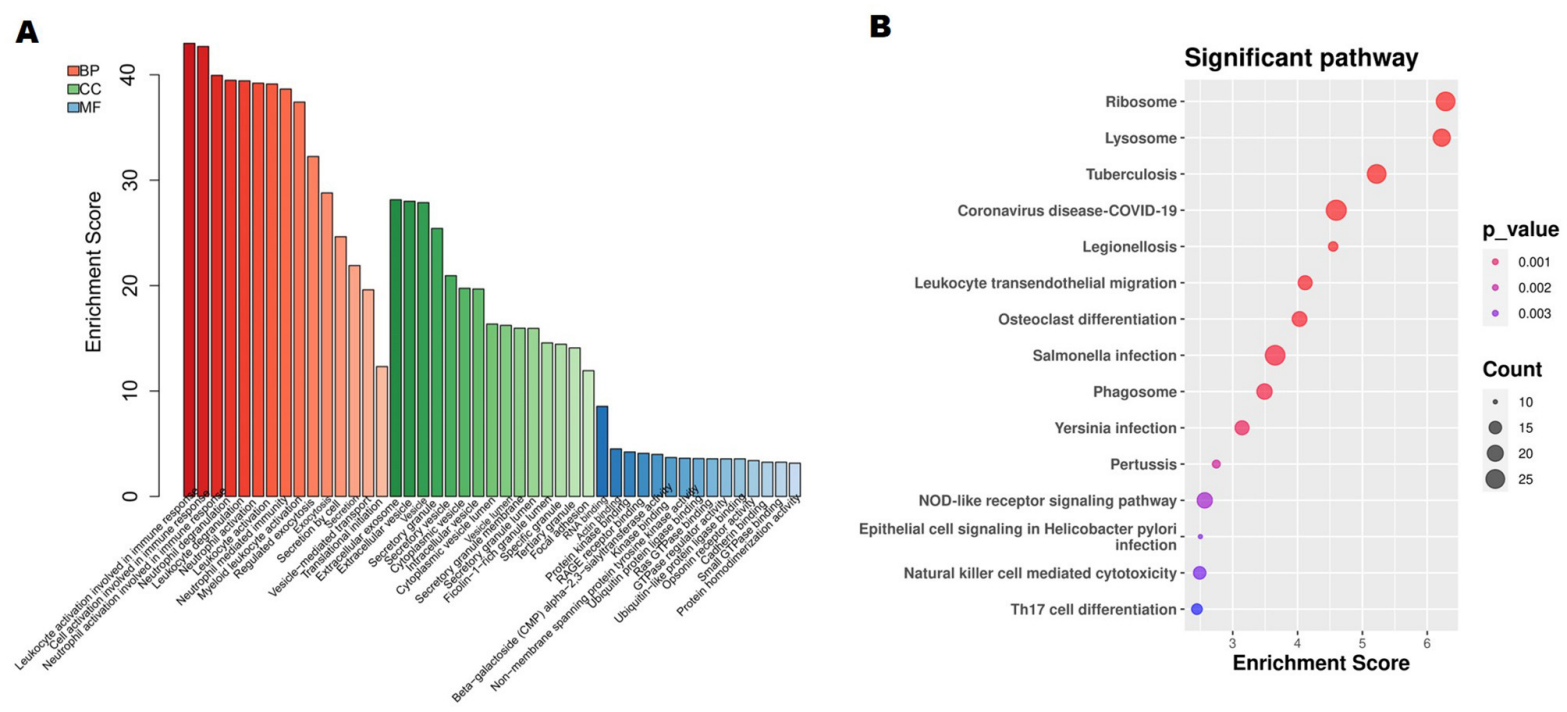
Analysis of differentially expressed genes' (DEGs) functional enrichment. **(A)** The top 15 functionally enriched important items according to gene ontology. Molecular function (MF); biological process (BP); cellular component (CC). **(B)** Kyoto Encyclopedia of Genes and Genomes analysis of the fifteen most consequential words.

We annotated the functions of the DEGs to further analyze their significance in anti-NMDAR encephalitis pathogenesis. Immune response-related leukocyte, cell, and neutrophil activations, as well as neutrophil degranulation and activation, were considerably enriched in the DEGs, according to an examination of GO terms (Figure 2A). Moreover, the ribosome, lysosome, tuberculosis, leukocyte transendothelial migration, and NOD-like receptor signaling pathways were the most significantly enriched pathways based on KEGG analysis (Figure 2B).

**Figure 3:**
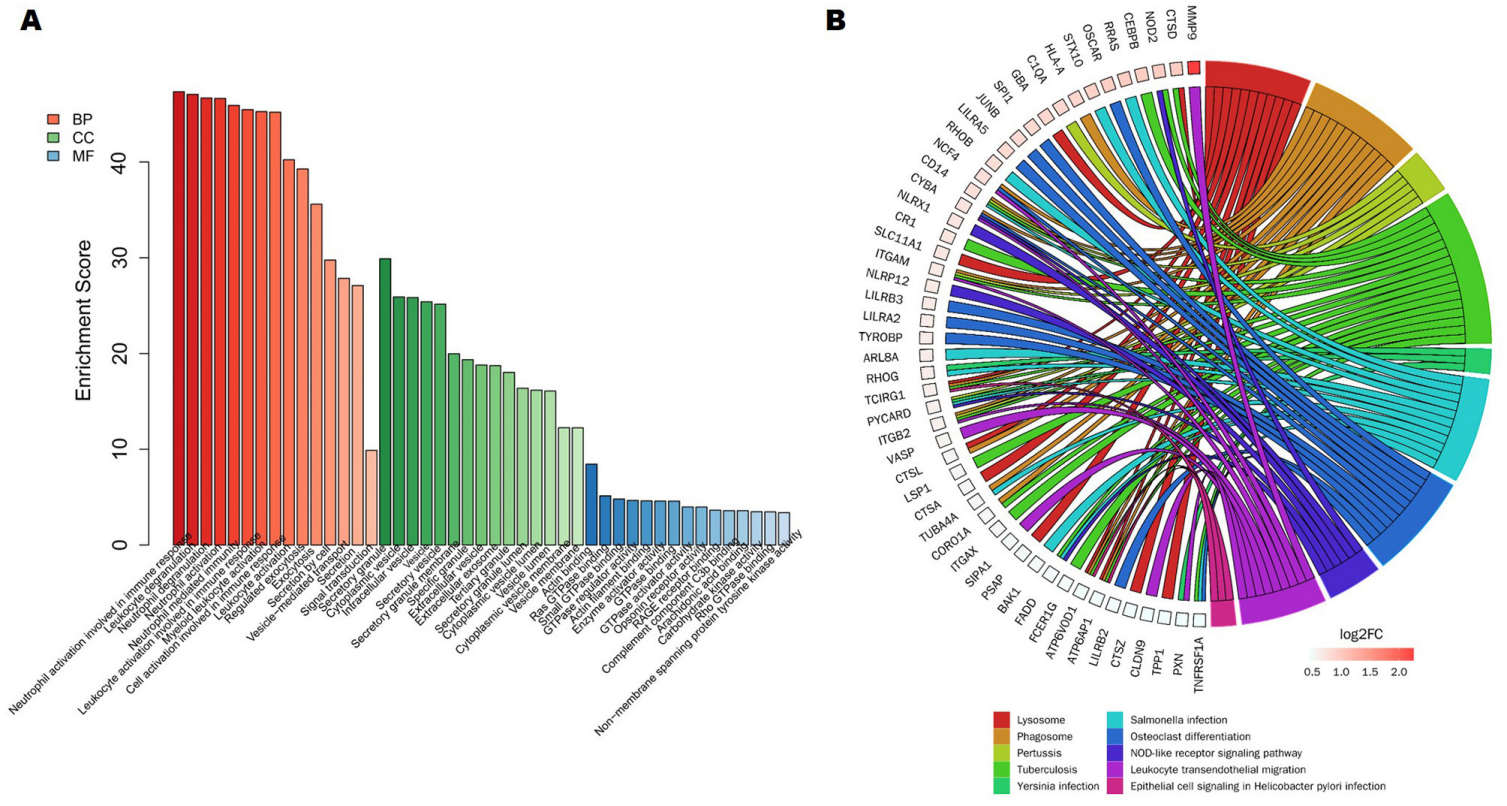
A study of 568 increased DEGs using functional enrichment. **(A)** The top 15 functionally enriched important items according to gene ontology. **(B)** The Kyoto Encyclopedia of Genes and Genomes study's DEGs related to the top 10 enriched pathways.

Further GO analysis revealed that 568 upregulated genes were enriched in multiple neutrophil mediated immune inflammatory processes, including neutral activation involved in immune response, neutral degranulation, and neutral activation (Figure 3A). Given our recent research findings implied that neutrophil extracellular traps (NETs) may play a role in the anti-NMDAR encephalitis process. Then, referring to 69 NET-related genes from previous studies (31), we screened 15 NET-related genes among 899 DEGs, including HMGB1, MMP9, and CSF3R (Supplementary Table 4). Additionally, Lysosome, Phagosome, Leukocyte transendothelial migration, and TNF signaling pathway were the remarkably enriched pathways based on KEGG analysis (Figure 3B).

**Figure 4:**
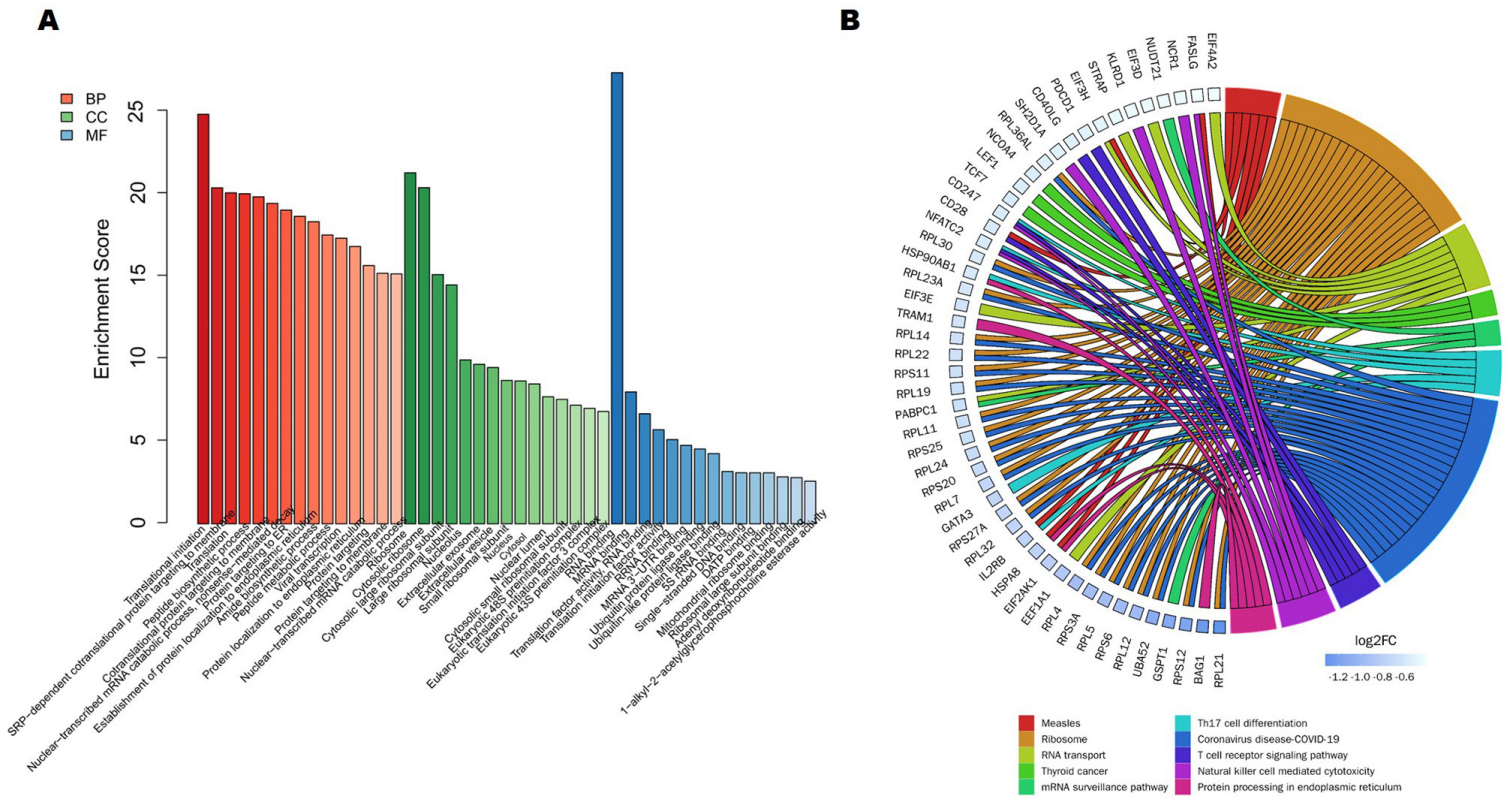
A study of 331 downregulated DEGs using functional enrichment. **(A)** The top 15 functionally enriched important items according to gene ontology. **(B)** The Kyoto Encyclopedia of Genes and Genomes study's DEGs related to the top 10 enriched processes.

331 downregulated genes GO analysis results showed that DEGs mainly enriched in pathways related to transcription and translation processes, such as translational initiation, nuclear transcribed mRNA catabolic process, and viral transcription (Figure 4A). Based on KEGG analysis, downregulated DEGs mainly enriched in Ribosome, RNA transport, Protein processing in endoplasmic reticulum, T cell receptor signaling pathway, and Th17 cell differentiation pathways (Figure 4B).

Functional enrichment analyses of 78 immune-related DEGs showed that biological processes related to inflammatory response, including response to cytokine, leukocyte activation, neutrophil degranulation, and T cell receptor signaling pathway pathways were enriched (Supplementary Figure 2), implying that immune dysregulation is involved in anti-NMDAR encephalitis pathogenesis.

**Figure 5:**
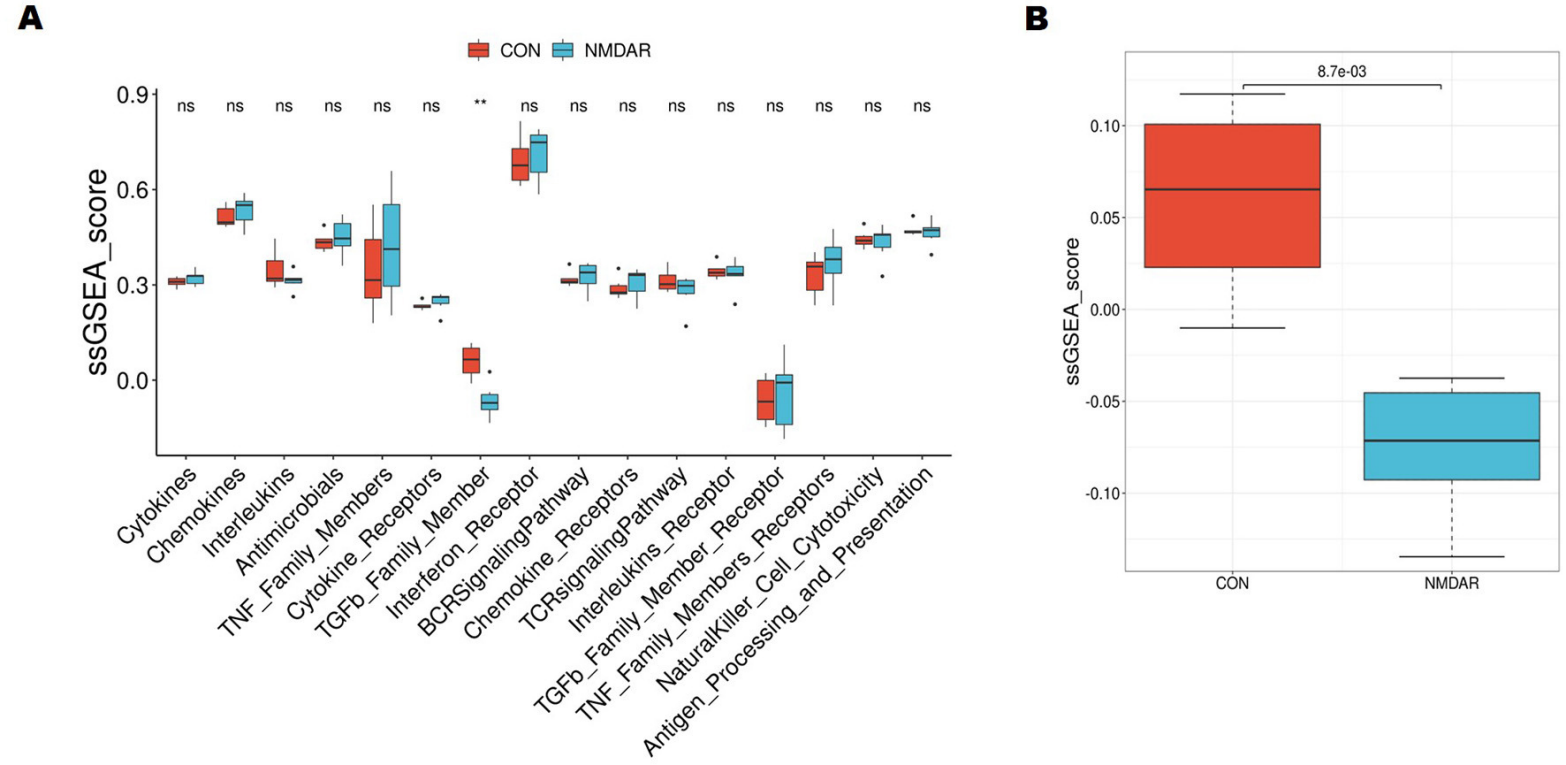
Single-sample gene set enrichment analysis (ssGSEA) analysis between anti- N-methyl-D-aspartate receptor (NMDAR) encephalitis patients and controls. **(A)** Boxplot of the signature scores for differential immune-related pathways. **(B)** The ssGSEA results present the scores of TGF-β family member genes between the anti-NMDAR encephalitis patients and controls. **p < 0.01.

Then, we further explored whether the control and anti-NMDAR encephalitis patients had different immune infiltration characteristics. Immune-related genes were quantified based on the ImmPort database using the GSVA R package. As ssGSEA results presented the scores of genes from the TGF-β family, member genes were significantly differentially expressed between the anti-NMDAR encephalitis patients and controls (*p* = 0.0087, FDR = 0.139) (Figures 5A, B).

### 3.3 Screening and verification of potential diagnostic biomarkers

**Figure 6:**
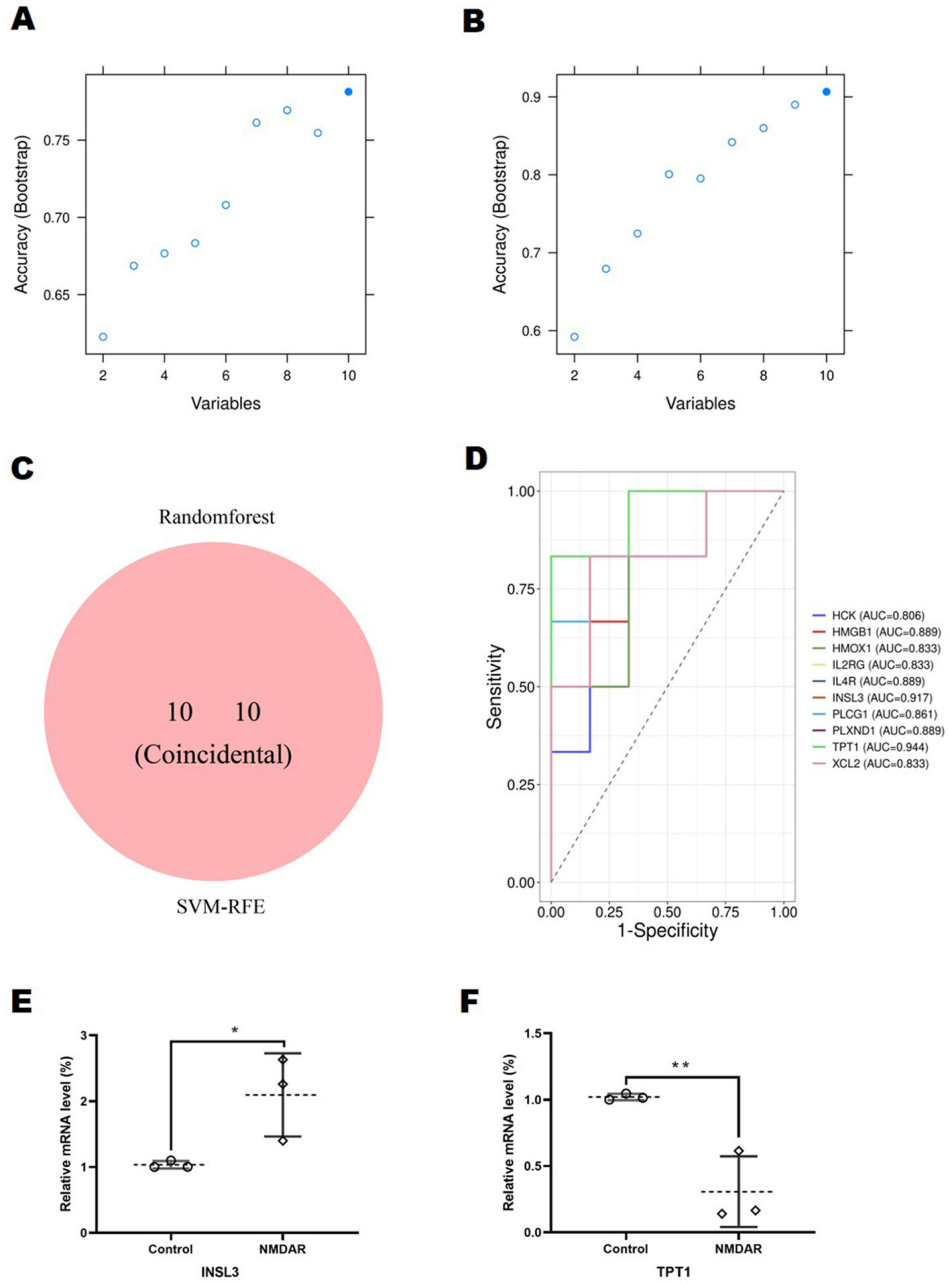
Identification and validation of diagnostic markers. Individuals with anti-N-methyl-D-aspartate receptor encephalitis showed higher levels of the 1–10 DEG prediction models in SVM-RFE **(B)** and random forest **(A)** models. **(C)** Venn diagram showing where the diagnostic markers obtained from the two approaches overlap. Support vector machine-recursive feature extraction, or SVM-RFE. **(D)** Receiver operating characteristic analysis-based diagnostic indicator diagnostic efficiency. The figure presents genes exhibiting area under the curve (AUC) values exceeding 0.8 in the receiver operating characteristic (ROC) analysis. Potential diagnostic signature expression was confirmed in **(E)** and **(F)** using quantitative real-time polymerase chain reaction (*n* = 3 in each case). INSL3 expression was considerably increased (*p* = 0.0441) in comparison with controls, whereas TPT1 expression declined considerably (*p* = 0.0099) in patients with anti-NMDAR encephalitis. ^*^p < 0.05, ^**^p < 0.01.

The RF algorithm was used in this study to discover ten important biomarkers from DEGs (Figure 6A). Using the SVM-RFE technique, ten genes were identified as potential DEG biomarkers (Figure 6B). The two groups of identified biomarkers included the consistent genes (Figure 6C), comprising six upregulated (hematopoietic cell kinase, heme oxygenase 1, interleukin 2 receptor subunit gamma, *interleukin 4 receptor*, insulin-like factor 3 [*INSL3*], and *PLXND1*) and four downregulated genes (tumor protein translationally controlled regulator 1 [*TPT1*], *HMGB1*, phospholipase C gamma 1, and X-C motif chemokine ligand 2). ROC curve analysis demonstrated that all ten genes achieved area under the curve (AUC) values exceeding 0.8, suggesting excellent diagnostic potential for anti-NMDAR encephalitis. Notably, *TPT1* and *INSL3* ROC curves demonstrated their potential as important biomarkers, with AUC values of 0.944 and 0.917, respectively (Figure 6D), showing strong predictive accuracy.

The expression levels of these two potential diagnostic biomarkers were examined using RT-qPCR. The results demonstrated consistency and reproducibility: INSL3 expression was considerably increased (*p* = 0.0441) in comparison with controls (Figure 6E), whereas TPT1 expression declined considerably (*p* = 0.0099) in patients with anti-NMDAR encephalitis (Figure 6F).

### 3.4 Infiltration of immune cells results

**Figure 7:**
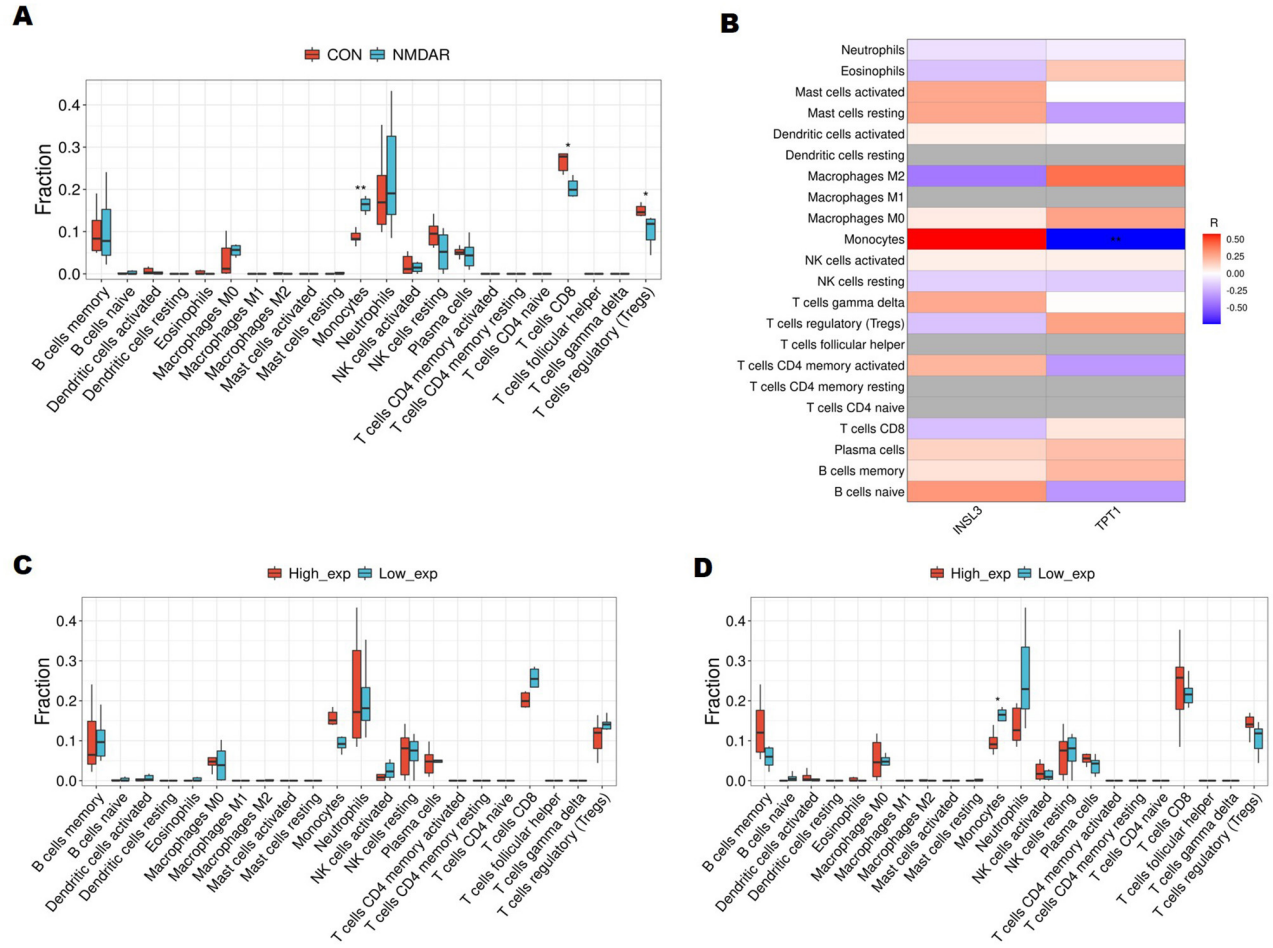
Immune Cell Composition in Peripheral Blood. **(A)** Boxplot showing differences in immune cell proportions between anti-NMDAR encephalitis patients and healthy controls. **(B)** The relationship between immune cell subsets and key genes (insulin-like factor 3 [*INSL3*] and tumor protein translationally controlled regulator 1 [*TPT1*]). Red indicates a positive association, whereas blue indicates a negative correlation. Darker colors imply a greater link. **(C)** Boxplot showing the infiltration of immune cells between *INSL3* groups with high and low expression. **(D)** Boxplot showing immune cell infiltration between *TPT1* expression groups with high and low levels *p < 0.05, **p < 0.01.

Using CIBERSORT, we aggregated the data from six healthy controls and six anti-NMDAR encephalitis patients to see if the immunological landscape of these individuals differed from that of control participants. Compared to control samples, anti-NMDAR encephalitis samples comprised a significantly higher proportion of monocytes, whereas the proportions of CD8^+^ T and T regulatory cells (Tregs) were significantly lower (*p* < 0.05) (Figure 7A). Furthermore, a correlation study showed that a negative relationship between TPT1 and resting mast cells (*R* = −0.741, *p* = 0.00578) (Figure 7B).

We divided the samples into high-expression and low-expression groups based on the expression levels of the two necessary genes for the model, INSL3 and TPT1. Between groups with high and low INSL3 expression, there was no discernible difference in immune cell infiltration (Figure 7C). On the other hand, there were significant differences in the number of monocytes between the groups expressing high and low levels of TPT1 (Figure 7D).

## 4 Discussion

Since being described by Dalmau et al. in 2007, anti-NMDAR encephalitis has been the most prevalent form of autoimmune encephalitis (1). Anti-NMDAR encephalitis' pathogenesis, diagnosis, and treatment have been studied recently. Anti-NMDAR encephalitis accompanied by autonomic dysfunction and related complications may affect long-term prognosis. EEG is gradually becoming more useful for predicting the long-term prognosis of patients (2, 5). Nevertheless, the research on biomarkers for the diagnosis and prognosis of anti-NMDAR encephalitis needs further exploration. Identifying this disease before auto-antibody detection results confirmed remains a challenge (5). We hypothesized in this study that immunoinflammation may be involved in the pathophysiology of anti-NMDAR encephalitis. Moreover, we identified two novel biomarkers candidates (*INSL3* and *TPT1*) that separated anti-NMDAR encephalitis patients from healthy controls.

Autoimmune encephalitis is most likely induced by antibodies attaching to neuronal surfaces; yet, the functions of additional immune effector pathways are mainly unknown (3). Potential causes (such as tumors or infections) have been hypothesized as predictors of abnormal immune activation in anti-NMDAR encephalitis (23). Autopsies of anti-NMDAR encephalitis patients demonstrated inflammatory cell infiltration into the brain's perivascular, interstitial, and Virchow-Robin areas (24). These findings showed that anti-NMDAR encephalitis is partly caused by immune system inflammation. Nevertheless, it is uncertain which specific immune effector pathways lead to anti-NMDAR encephalitis. Our findings reveal that DEGs are enriched in a variety of immunoregulatory processes, including leukocyte activation implicated in immunological response, Th17 cell differentiation, and Th1 and Th2 cell differentiation. Immunological cell infiltration also suggested that the immune landscapes of anti-NMDAR encephalitis differed from that of control participants. These results broaden the body of data supporting the hypothesis that anti-NMDAR encephalitis is partly caused by immune system inflammation.

Specifically, functional enrichment analysis revealed that DEGs were more likely to be involved in biological processes that involve neutrophils, including immune response activation, degranulation, and activation. Previous research (3, 25) indicates that immune system abnormalities mediated by B lymphocytes play a significant role in the pathogenesis of anti-NMDAR encephalitis. A large number of B cells and CD138+antibody secreting cells were observed infiltrating the brain tissue of patients with anti NMDAR encephalitis during autopsy. Wagnon et al. (22) found in an animal model of anti NMDAR encephalitis B-cell response can lead to an autoimmune reaction against NMDAR that drives encephalitis-like behavioral impairments. However, the involvement of neutrophils in this disease has not been thoroughly investigated. As the initial line of defense against pathogen invasion, neutrophils play a crucial role in the immune response (26). Moreover, they have been found to be involved in a number of diseases of the neurological system, such as encephalitis (27), Alzheimer's disease (28), and venous sinus thrombosis (29). Our new research (30) indicates that neutrophil extracellular traps (NETs) can be produced by peripheral blood neutrophils from patients with anti-NMDAR encephalitis, suggesting a potential function for NETs in this pathological process. In this study, referring to 69 NET-related genes from previous studies (31), we screened 15 NET-related genes among 899 DEGs, such as *HMGB1, MMP9*, and *PADI4* (data not shown). Functional enrichment analysis showed that inflammation related pathways such as neutrophil activation and leukocyte migration were significantly activated, suggesting that NETs may participate in disease progression through key genes such as HMGB1 and MMP9. This discovery provides a new research direction for the immunopathological mechanism of anti NMDAR encephalitis. This will offer insights into how neutrophils function in the immune system's control of the illness. To precisely grasp the underlying mechanism of action, additional study is necessary.

In order to better understand the immune systems that control the course of the illness and to establish predictors of long-term outcomes, considerable efforts have recently been made to find biomarkers. Most of these biomarkers, meanwhile, are still in the investigation phase. More research is needed to find and validate new biomarkers for clinical decision-making. In the present study, we also identified two potential biomarkers (*INSL3* and *TPT1*) candidates that distinguish anti-NMDAR encephalitis patients from controls. *INSL3* is a member of the relaxin family (32). Previous research has suggested that it has an important function in cancers and reproductive system illnesses (33, 34). However, research on its role in neuroimmune regulation is limited, and no research has suggested that it plays a role in anti-NMDAR encephalitis. Intriguingly, our finding of elevated INSL3 in anti-NMDAR encephalitis, indicating a role of INSL3 in the regulation of neuroinflammation. In addition, it also suggests potential cross-talk between reproductive hormones and neuroinflammation, which may provide clues for exploring its role in anti-NMDAR encephalitis. *TPT1* is the gene that encodes the translationally controlled tumor protein (TCTP) (35). Many biological processes, including cell proliferation and differentiation, immunological control, and tumor development, have been demonstrated to require TCTP (35, 36). A study suggested that diminution of *TPT1* up-regulates miR-200a to improve neurobehavior and oxidative stress injury in cerebral palsy rats (37). Another study showed that *TPT1* increased after spinal cord injury, and *TPT1* might play a crucial role in astrocyte proliferation and migration. regulation of apoptotic process, regulation of intracellular signal transduction (38). Our study's enrichment analysis results indicate that TPT1 primarily functions in biological processes, including internal signal transmission and apoptotic process control. We are now investigating the pathophysiology of anti-NMDAR encephalitis, which might offer new insights into the origins and course of the illness. Future studies should incorporate longitudinal sampling across distinct disease phases (acute, remission, and relapse) to determine whether INSL3 and TPT1 exhibit stage-specific expression patterns. Additionally, integrating single-cell sequencing to resolve time-dependent immune cell subset dynamics could elucidate the regulatory mechanisms underlying biomarker fluctuations.

This work characterized the immune cell infiltration features and the transcriptome-wide landscapes of mRNAs in anti-NMDAR encephalitis. However, it has certain limitations. This disease is uncommon, and the study's tiny sample size represents a constraint. Due to the limited sample size, this study may be subject to selection biases, including but not limited to age, gender, and geographical heterogeneity. Future multicenter studies with larger cohorts are warranted to evaluate the influence of age stratification, disease progression stages, and therapeutic regimens on biomarker expression profiles. As for the diagnostic performance shown with ROC curve, one should notice that the validation samples are from the same datasets. Considering that anti-NMDAR encephalitis shows high heterogeneities in clinical manifestations, the diagnostic performance of biomarkers screened in this study should be tested with more samples. Therefore, we will collect anti-NMDAR encephalitis samples, and test the diagnostic performance of potential biomarkers in the future. In addition, further verification using cellular and animal experiments may provide more evidence to clarify the functions of these two key genes in anti-NMDAR encephalitis.

## 5 Conclusion

This study systematically elucidates the molecular characteristics and potential regulatory mechanisms of the peripheral immune microenvironment in anti-NMDAR encephalitis by integrating transcriptomic sequencing and immunoinformatic analyses. We report for the first time the diagnostic value of INSL3 and TPT1 in anti NMDAR encephalitis and reveal the potential pathogenic role of monocyte T-cell axis imbalance. These findings not only expand our understanding of the molecular mechanisms of diseases, but also provide new ideas for developing targeted immunomodulatory therapies.

## Data Availability

The original contributions presented in the study are included in the article/Supplementary material, further inquiries can be directed to the corresponding authors.

## Supplementary material

Supplementary Figure 1 A heatmap containing all 899 DEGs comparing individuals suffering from anti-NMDAR encephalitis to controls.

Supplementary Figure 2 Functional enrichment study of 78 DEGs associated with immunity. **(A)** The top 15 functionally enriched important items according to gene ontology. **(B)** Kyoto Encyclopedia of Genes and Genomes analysis of the fifteen most consequential words.

Supplementary Table 1 Primer sequences for Quantitative real-time PCR.

Supplementary Table 2 Results of differential expression.

Supplementary Table 3 78 overlapping genes.

Supplementary Table 4 NET-related genes among 899 differentially expressed genes.
