# Supplementary figures and images for: Immune inflammatory regulation in Anti-NMDAR encephalitis: insights from transcriptome analysis
Source: Front Neurol. 2025 May 9;16:1568274. doi: 10.3389/fneur.2025.1568274 (PMC12098042; doi:10.3389/fneur.2025.1568274)

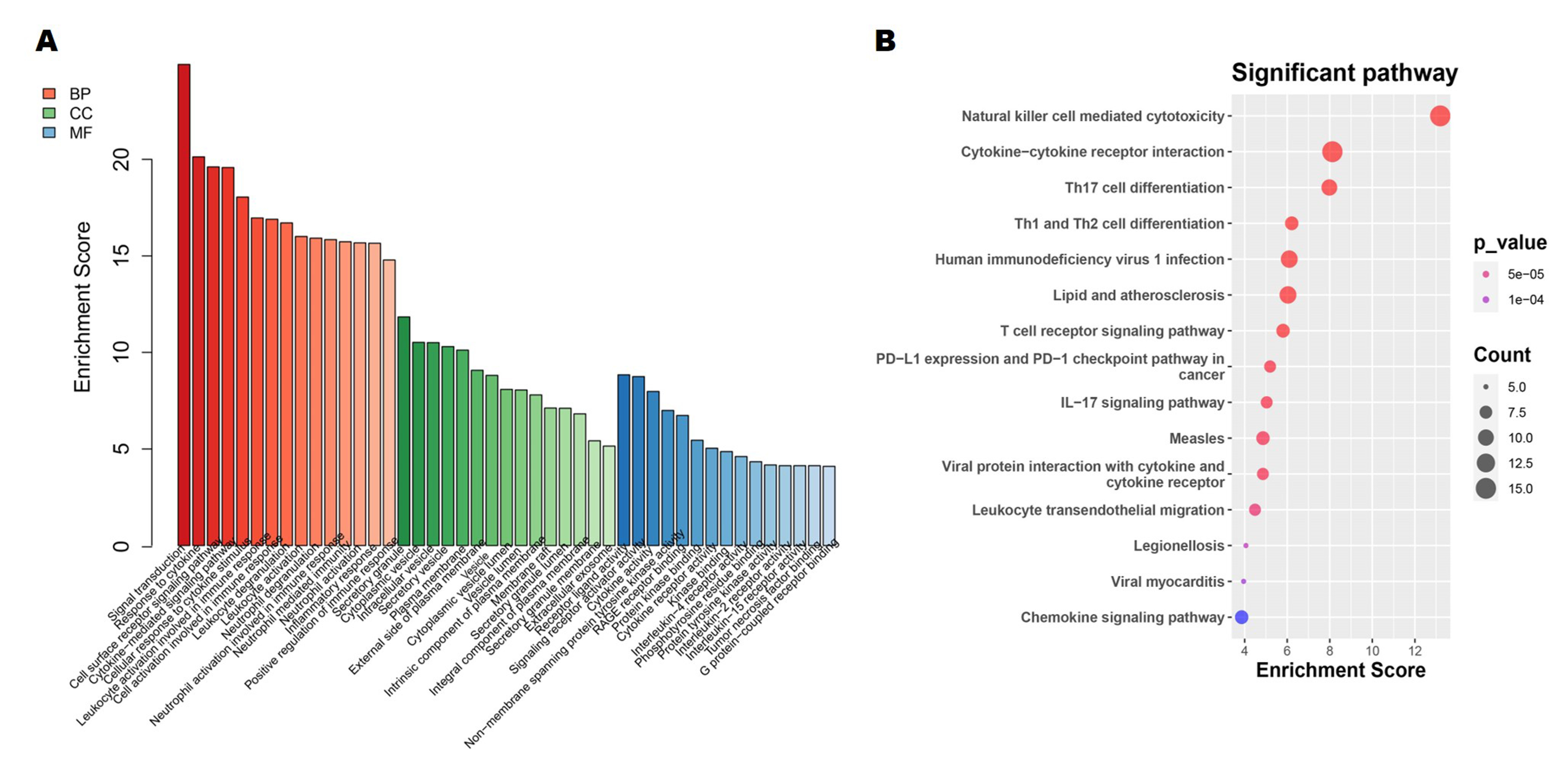

Supplement: Supplementary Figure 2 — Functional enrichment study of 78 DEGs associated with immunity. (A) The top 15 functionally enriched important items according to gene ontology. (B) Kyoto Encyclopedia of Genes and Genomes analysis of the fifteen most consequential words. [file Image_2.jpeg]
