# Supplementary material for: Immune inflammatory regulation in Anti-NMDAR encephalitis: insights from transcriptome analysis
Source: Front Neurol. 2025 May 9;16:1568274. doi: 10.3389/fneur.2025.1568274 (PMC12098042; doi:10.3389/fneur.2025.1568274)
Supplement: Supplementary Table 1 — Primer sequences for Quantitative real-time PCR. [file Table_1.docx]

Supplementary Table 1 Primer sequences for Quantitative real-time PCR

| Target | Gene ID | Forward 5’→3’ | Reverse 5’→3’ |
| --- | --- | --- | --- |
| NSL3 | 55683 | CCACTCTATGACAATCAGAAGGC | GGGGCATCTGCATCAGTCC |
| TPT1 | 7178 | GAAAGCACAGTAATCACTGGTGT | GCAGCCCCTGTCATAAAAGGT |
| GAPDH | 2597 | GCACCGTCAAGGCTGAGACC | ATGGTGGTGAAGACGCCAGT |
