# Supplementary material for: Immune inflammatory regulation in Anti-NMDAR encephalitis: insights from transcriptome analysis
Source: Front Neurol. 2025 May 9;16:1568274. doi: 10.3389/fneur.2025.1568274 (PMC12098042; doi:10.3389/fneur.2025.1568274)
Supplement: Supplementary Table 2 — Results of differential expression. [file Table_2.docx]

**Supplementary Table 2 Results of Differential Expression**

| **Gene_Name** | **log2FC** | **FC** | **p_value** | **p. Adjust** | **NMDAR** | **CON** |
| --- | --- | --- | --- | --- | --- | --- |
| MMP9 | 2.253988479 | 4.769997397 | 0.006072932 | 0.184509368 | 7.172075954 | 4.918087475 |
| RETN | 2.199851386 | 4.594320129 | 0.02061646 | 0.265908505 | 4.972521885 | 2.772670499 |
| SLPI | 1.74029617 | 3.341037486 | 0.006868453 | 0.188985286 | 4.937173834 | 3.196877664 |
| PADI4 | 1.4588108 | 2.748816878 | 0.000515085 | 0.101895286 | 4.898334727 | 3.439523928 |
| RGL4 | 1.346836447 | 2.543537645 | 0.010774258 | 0.212867508 | 2.696447252 | 1.349610805 |
| CDA | 1.344333312 | 2.53912833 | 0.001066183 | 0.124883735 | 6.719915411 | 5.375582099 |
| CCDC71L | 1.334580083 | 2.52202064 | 0.015012573 | 0.238646297 | 3.15665251 | 1.822072428 |
| HK3 | 1.266096904 | 2.405100039 | 0.000248234 | 0.076199348 | 5.617292168 | 4.351195264 |
| HIST1H2AE | 1.20863413 | 2.311187218 | 0.01516608 | 0.238946208 | 3.503701816 | 2.295067685 |
| DUSP1 | 1.169017345 | 2.248584881 | 0.038811135 | 0.33076938 | 7.218768053 | 6.049750708 |
| CA4 | 1.164431908 | 2.241449363 | 0.009754653 | 0.206752197 | 2.792785104 | 1.628353196 |
| HIST1H4H | 1.164249745 | 2.241166363 | 0.010471782 | 0.209954512 | 2.112119243 | 0.947869498 |
| FUT7 | 1.118038899 | 2.170517266 | 0.004842575 | 0.175953494 | 3.79800644 | 2.679967541 |
| MCEMP1 | 1.113483586 | 2.163674661 | 0.01445207 | 0.235755583 | 2.914152447 | 1.800668861 |
| CKAP4 | 1.098853356 | 2.141843925 | 0.001050207 | 0.124883735 | 4.125368134 | 3.026514779 |
| OPRL1 | 1.068021367 | 2.096556002 | 0.001858021 | 0.143432071 | 2.720826766 | 1.652805399 |
| TCN2 | 1.059864738 | 2.084736055 | 0.002427427 | 0.15111156 | 2.349805675 | 1.289940937 |
| ASPRV1 | 1.029583444 | 2.041434733 | 0.000647153 | 0.109965644 | 2.369674936 | 1.340091492 |
| KLHDC8B | 1.025699936 | 2.035946902 | 0.011793309 | 0.22052893 | 3.048800641 | 2.023100705 |
| FCN1 | 1.023088419 | 2.032264834 | 7.25414E-07 | 0.006457638 | 6.999382882 | 5.976294463 |
| ST14 | 1.020015289 | 2.02794045 | 0.002114763 | 0.147692828 | 2.400690302 | 1.380675013 |
| TP53I11 | 1.012287904 | 2.017107404 | 1.06411E-05 | 0.031575646 | 3.169300281 | 2.157012376 |
| HSPA7 | 1.003251129 | 2.004512104 | 0.022434328 | 0.271508961 | 2.371670763 | 1.368419634 |
| ADGRG3 | 0.987079803 | 1.982168768 | 0.003469444 | 0.160859338 | 5.472207269 | 4.485127465 |
| CTSD | 0.984202458 | 1.978219423 | 0.005994569 | 0.184509368 | 6.608590602 | 5.624388144 |
| AC145098.1 | 0.98400574 | 1.977949702 | 0.003400886 | 0.160203084 | 4.626572914 | 3.642567174 |
| FHL3 | 0.982029766 | 1.975242477 | 0.002268174 | 0.147692828 | 4.269013279 | 3.286983512 |
| ALOX5AP | 0.979224434 | 1.971405334 | 0.009651113 | 0.206584049 | 6.751925605 | 5.772701171 |
| DYSF | 0.975447864 | 1.966251497 | 0.000219279 | 0.072473812 | 5.456809864 | 4.481362001 |
| NOD2 | 0.973299187 | 1.963325241 | 0.005688051 | 0.183035808 | 2.655683757 | 1.68238457 |
| CEBPB | 0.969651971 | 1.958368111 | 0.001361574 | 0.133695019 | 7.500066673 | 6.530414701 |
| RRAS | 0.96229705 | 1.948409672 | 0.001417502 | 0.135683908 | 3.56279573 | 2.60049868 |
| OSCAR | 0.960398948 | 1.945847905 | 0.001691397 | 0.143432071 | 5.369036499 | 4.408637551 |
| S100A8 | 0.95505419 | 1.938652452 | 0.025327179 | 0.281828184 | 9.852681073 | 8.897626882 |
| AGTRAP | 0.948879772 | 1.930373174 | 0.00179831 | 0.143432071 | 5.745425641 | 4.796545869 |
| PGD | 0.944751307 | 1.924857049 | 0.001289234 | 0.133695019 | 6.070319188 | 5.125567882 |
| LTB4R | 0.938441007 | 1.916456175 | 0.003356477 | 0.160203084 | 4.030246769 | 3.091805761 |
| KCNH3 | 0.938086801 | 1.915985711 | 0.019729341 | 0.263246608 | 1.670547026 | 0.732460225 |
| TBKBP1 | 0.931734094 | 1.907567481 | 0.00523314 | 0.179366897 | 3.371775745 | 2.440041651 |
| GRN | 0.92141407 | 1.893970776 | 0.001434958 | 0.135893528 | 7.562614582 | 6.641200511 |
| SLC16A3 | 0.918033878 | 1.889538453 | 0.002162129 | 0.147692828 | 4.831138962 | 3.913105084 |
| EPHB4 | 0.915982569 | 1.886853705 | 0.006266746 | 0.185955257 | 1.765709786 | 0.849727216 |
| STX10 | 0.915204627 | 1.885836533 | 0.005238755 | 0.179366897 | 5.167806324 | 4.252601697 |
| PADI2 | 0.912825389 | 1.882729047 | 0.008718699 | 0.201072176 | 3.967775731 | 3.054950341 |
| MGAM | 0.903157788 | 1.870154924 | 0.008856433 | 0.202673442 | 3.609660443 | 2.706502655 |
| S100A9 | 0.899962338 | 1.86601727 | 0.021072589 | 0.266811776 | 11.97491437 | 11.07495203 |
| B3GNT8 | 0.886000158 | 1.848045348 | 0.002754792 | 0.156159451 | 4.349925157 | 3.463924999 |
| PANX2 | 0.874206164 | 1.83299921 | 0.012363432 | 0.2225944 | 3.713741514 | 2.839535349 |
| HLA-A | 0.86974123 | 1.827335109 | 0.004301396 | 0.170726769 | 9.106344279 | 8.23660305 |
| UBTD1 | 0.868452751 | 1.825703835 | 0.018654736 | 0.258525185 | 3.66571568 | 2.797262929 |
| PEAK3 | 0.866739044 | 1.823536459 | 0.000875703 | 0.121855199 | 3.532719096 | 2.665980052 |
| MMP25 | 0.865109096 | 1.821477402 | 0.00392294 | 0.164726463 | 6.590954822 | 5.725845726 |
| DOK3 | 0.863812539 | 1.819841166 | 0.002525647 | 0.15128476 | 5.446485575 | 4.582673036 |
| S100A6 | 0.857451623 | 1.811835056 | 0.00512611 | 0.177558863 | 8.75157819 | 7.894126568 |
| C1QA | 0.855777833 | 1.809734215 | 0.005716014 | 0.183035808 | 2.597319058 | 1.741541225 |
| GBA | 0.854049611 | 1.807567611 | 0.000348921 | 0.083896316 | 3.290065919 | 2.436016308 |
| MTX1P1 | 0.851576988 | 1.804472289 | 0.018877662 | 0.259475892 | 2.448902634 | 1.597325646 |
| PFKFB3 | 0.850592711 | 1.80324161 | 0.020207759 | 0.264653675 | 3.187814207 | 2.337221496 |
| MSRB1 | 0.845872179 | 1.797351004 | 0.003685291 | 0.164726463 | 7.131029643 | 6.285157463 |
| SPI1 | 0.84387507 | 1.794864668 | 0.001157802 | 0.127294316 | 8.556474679 | 7.712599609 |
| F5 | 0.832421692 | 1.780671871 | 0.020216187 | 0.264653675 | 2.165928716 | 1.333507024 |
| AGPAT2 | 0.831583441 | 1.779637545 | 0.0178728 | 0.253547252 | 4.388872616 | 3.557289175 |
| JUNB | 0.829034325 | 1.776495858 | 0.009291928 | 0.205252455 | 7.560650735 | 6.731616411 |
| UPP1 | 0.824923157 | 1.771440683 | 0.001904624 | 0.143432071 | 2.977845188 | 2.152922031 |
| LILRA5 | 0.824274689 | 1.770644627 | 0.011848429 | 0.22052893 | 4.69003353 | 3.865758841 |
| QSOX1 | 0.821113523 | 1.766769121 | 0.001830177 | 0.143432071 | 3.118297966 | 2.297184443 |
| VAMP5 | 0.809059048 | 1.752068339 | 0.013343132 | 0.231158533 | 4.631740885 | 3.822681836 |
| IFITM2 | 0.808240813 | 1.751074922 | 0.003408685 | 0.160203084 | 10.3166954 | 9.508454588 |
| HOMER3 | 0.806036053 | 1.748400933 | 0.021641997 | 0.2673433 | 1.926378553 | 1.1203425 |
| ZYX | 0.805786767 | 1.748098849 | 0.001156712 | 0.127294316 | 7.548915595 | 6.743128829 |
| RHOB | 0.790250375 | 1.729374564 | 0.011817976 | 0.22052893 | 5.623990807 | 4.833740432 |
| PDLIM7 | 0.789008096 | 1.727886071 | 0.00299419 | 0.156159451 | 4.867106971 | 4.078098875 |
| NCF4 | 0.782507119 | 1.720117501 | 0.001804264 | 0.143432071 | 6.163180221 | 5.380673102 |
| CD14 | 0.776050142 | 1.712436085 | 5.55874E-05 | 0.072473812 | 7.439955734 | 6.663905591 |
| IER2 | 0.77246351 | 1.708184147 | 0.016162996 | 0.241740542 | 4.676860584 | 3.904397075 |
| AC018638.1 | 0.772253437 | 1.707935434 | 0.008517398 | 0.199237174 | 1.746240053 | 0.973986617 |
| OR52K3P | 0.766989704 | 1.701715314 | 0.03924003 | 0.332048235 | 2.343293019 | 1.576303315 |
| CMTM2 | 0.764312319 | 1.698560162 | 0.020864015 | 0.266811776 | 5.395917504 | 4.631605185 |
| PHC2 | 0.762591558 | 1.696535428 | 0.042491378 | 0.343558805 | 5.953904339 | 5.191312781 |
| P2RX1 | 0.760536882 | 1.694120955 | 0.000158666 | 0.072473812 | 3.12732677 | 2.366789888 |
| ADM | 0.758614141 | 1.691864631 | 0.007221908 | 0.191444492 | 3.635867401 | 2.87725326 |
| CEBPA | 0.755890032 | 1.688673052 | 0.046168604 | 0.357696183 | 3.40771027 | 2.651820238 |
| QPCT | 0.755772855 | 1.688535902 | 0.043577541 | 0.346768856 | 4.06336505 | 3.307592195 |
| AP5B1 | 0.751408047 | 1.683435034 | 0.040473721 | 0.336368149 | 4.960489818 | 4.209081771 |
| CYBA | 0.748040053 | 1.679509613 | 0.024284171 | 0.278282326 | 7.720147134 | 6.972107081 |
| SNAI3 | 0.740961068 | 1.671288817 | 0.003533784 | 0.161961772 | 3.390049798 | 2.64908873 |
| ATP6V0B | 0.740421605 | 1.670663993 | 0.03842695 | 0.329762578 | 5.498766707 | 4.758345102 |
| GALK1 | 0.732181679 | 1.661149224 | 0.043671227 | 0.346768856 | 3.325934181 | 2.593752502 |
| SERPINA1 | 0.727969106 | 1.656305854 | 0.042347013 | 0.343558805 | 6.974655302 | 6.246686195 |
| DDAH2 | 0.723578727 | 1.651273081 | 0.000193405 | 0.072473812 | 3.185273107 | 2.46169438 |
| SLC2A3 | 0.722237376 | 1.649738517 | 0.024064181 | 0.27757182 | 4.918760624 | 4.196523248 |
| NLRX1 | 0.721545962 | 1.648948066 | 0.02814209 | 0.295553315 | 2.523017649 | 1.801471688 |
| LGALS1 | 0.720595367 | 1.647861929 | 0.005774141 | 0.18357645 | 6.645419702 | 5.924824335 |
| NATD1 | 0.720422508 | 1.647664499 | 0.017939777 | 0.253547252 | 4.076129872 | 3.355707364 |
| CAPG | 0.71946755 | 1.646574228 | 0.002587927 | 0.152567726 | 5.000274828 | 4.280807278 |
| CR1 | 0.714896899 | 1.641365914 | 0.003816093 | 0.164726463 | 2.973990653 | 2.259093755 |
| FRAT2 | 0.712254252 | 1.638362106 | 0.023151184 | 0.273813724 | 6.742810545 | 6.030556292 |
| ZFP36 | 0.710929853 | 1.636858773 | 0.017578952 | 0.252807482 | 7.008727751 | 6.297797898 |
| CHST13 | 0.709441757 | 1.635171274 | 0.040620754 | 0.336368149 | 2.788609286 | 2.079167529 |
| TMEM120A | 0.702665227 | 1.627508668 | 0.020123117 | 0.264653675 | 4.394569388 | 3.69190416 |
| LST1 | 0.701005262 | 1.625637133 | 0.001856235 | 0.143432071 | 5.806108223 | 5.10510296 |
| GPR27 | 0.700271382 | 1.624810404 | 0.037940073 | 0.328109269 | 3.065814909 | 2.365543526 |
| SLC11A1 | 0.700225882 | 1.624759161 | 0.04570459 | 0.355959979 | 4.567995137 | 3.867769255 |
| PLPPR2 | 0.69950429 | 1.623946709 | 0.010785577 | 0.212867508 | 5.098347472 | 4.398843182 |
| FPR1 | 0.698787651 | 1.623140236 | 0.008571988 | 0.199237174 | 8.100992585 | 7.402204934 |
| PLIN3 | 0.698146561 | 1.622419122 | 0.001343795 | 0.133695019 | 4.373522262 | 3.675375701 |
| APMAP | 0.696450519 | 1.620512915 | 0.012027564 | 0.221502022 | 5.575291659 | 4.87884114 |
| PRAM1 | 0.696164849 | 1.620192068 | 0.004978833 | 0.176488433 | 4.276313763 | 3.580148914 |
| IMPDH1 | 0.695898468 | 1.61989294 | 0.013963452 | 0.23374783 | 5.668879387 | 4.972980919 |
| FES | 0.695598981 | 1.619556704 | 0.009572404 | 0.206584049 | 4.322916139 | 3.627317158 |
| FLOT1 | 0.695122749 | 1.619022179 | 0.000223159 | 0.072473812 | 5.130859314 | 4.435736565 |
| SPATA2L | 0.694547336 | 1.618376567 | 0.001026973 | 0.124883735 | 2.959049644 | 2.264502309 |
| APOBR | 0.694153458 | 1.617934786 | 0.006179199 | 0.185530272 | 5.917495257 | 5.223341799 |
| RASGRP4 | 0.692148657 | 1.615688029 | 0.002895608 | 0.156159451 | 5.239074308 | 4.54692565 |
| LRRC25 | 0.686208305 | 1.60904905 | 0.008348926 | 0.199237174 | 6.744806628 | 6.058598322 |
| ITGAM | 0.686054795 | 1.608877847 | 0.000131901 | 0.072473812 | 5.215033711 | 4.528978916 |
| S100A11 | 0.684342027 | 1.606968921 | 0.008981766 | 0.203391487 | 9.852823491 | 9.168481464 |
| RGS14 | 0.683359833 | 1.605875261 | 0.019753846 | 0.263246608 | 5.076617006 | 4.393257173 |
| NLRP12 | 0.682745582 | 1.605191679 | 0.01182763 | 0.22052893 | 3.63042948 | 2.947683898 |
| NBEAL2 | 0.681200983 | 1.603474025 | 0.014334517 | 0.235755583 | 5.183055672 | 4.501854689 |
| TSEN34 | 0.676758118 | 1.598543627 | 0.016989361 | 0.247527479 | 4.840114827 | 4.163356708 |
| CYSTM1 | 0.676699133 | 1.598478271 | 0.039619445 | 0.333042776 | 3.283041505 | 2.606342372 |
| S100A4 | 0.675751423 | 1.597428572 | 0.01562104 | 0.239956533 | 8.408758517 | 7.733007093 |
| PLEKHO2 | 0.675046529 | 1.596648266 | 0.001836733 | 0.143432071 | 5.780393378 | 5.105346849 |
| ALDOA | 0.674676976 | 1.59623933 | 0.014298905 | 0.235755583 | 2.355304992 | 1.680628015 |
| C19orf38 | 0.673549201 | 1.594992014 | 0.012052471 | 0.221502022 | 5.372883444 | 4.699334243 |
| FLOT2 | 0.67098319 | 1.592157646 | 0.036608792 | 0.323626082 | 7.042013922 | 6.371030732 |
| BST1 | 0.666293536 | 1.586990548 | 0.028578377 | 0.297044096 | 3.860122841 | 3.193829305 |
| DHX34 | 0.666251093 | 1.586943862 | 0.00145788 | 0.136271371 | 3.181932337 | 2.515681243 |
| TOM1 | 0.664786715 | 1.585333884 | 0.008489153 | 0.199237174 | 3.472999033 | 2.808212318 |
| LILRB3 | 0.663809567 | 1.584260488 | 0.004079208 | 0.166573898 | 5.243619766 | 4.579810199 |
| TBC1D2 | 0.660931506 | 1.581103167 | 0.005588839 | 0.183035808 | 2.704656719 | 2.043725213 |
| LILRA2 | 0.65840194 | 1.57833335 | 0.004753482 | 0.175385196 | 4.857665354 | 4.199263414 |
| TYROBP | 0.657052564 | 1.5768578 | 0.011019183 | 0.214645011 | 8.734104513 | 8.077051949 |
| ZBTB7B | 0.656191163 | 1.575916573 | 0.003419297 | 0.160203084 | 5.405631235 | 4.749440073 |
| ZNF787 | 0.65577811 | 1.575465443 | 0.006973415 | 0.189260177 | 3.656664342 | 3.000886231 |
| DEF8 | 0.65575988 | 1.575445536 | 0.006379646 | 0.186909523 | 3.730211653 | 3.074451773 |
| ROPN1L | 0.654900553 | 1.574507416 | 0.000209837 | 0.072473812 | 2.094960406 | 1.440059854 |
| GNG5 | 0.65467389 | 1.574260063 | 0.001366687 | 0.133695019 | 2.959897478 | 2.305223588 |
| SOWAHD | 0.654518807 | 1.574090848 | 0.0263079 | 0.286690482 | 2.878326568 | 2.22380776 |
| NDST1 | 0.65179538 | 1.571122182 | 0.020623324 | 0.265908505 | 2.699730117 | 2.047934738 |
| SEMA4A | 0.651203377 | 1.570477612 | 0.002910778 | 0.156159451 | 4.07035237 | 3.419148993 |
| MBOAT7 | 0.650275728 | 1.569468124 | 0.00438121 | 0.171059357 | 6.847751776 | 6.197476048 |
| VSIR | 0.649822039 | 1.568974646 | 0.024820433 | 0.279850937 | 7.482735896 | 6.832913857 |
| GPSM3 | 0.648662651 | 1.567714282 | 0.021186124 | 0.266811776 | 7.810028545 | 7.161365894 |
| TKT | 0.647200554 | 1.56612629 | 0.005341722 | 0.179567556 | 6.056380451 | 5.409179897 |
| RENBP | 0.644281845 | 1.562961071 | 0.026938812 | 0.288794919 | 3.395527843 | 2.751245998 |
| SLC16A5 | 0.640174916 | 1.558518107 | 0.021110415 | 0.266811776 | 2.352682762 | 1.712507846 |
| ARL8A | 0.639706072 | 1.558011705 | 0.000353173 | 0.083896316 | 3.972247002 | 3.33254093 |
| LPAR2 | 0.638519869 | 1.556731213 | 0.006001145 | 0.184509368 | 3.772838632 | 3.134318763 |
| RHOG | 0.636790505 | 1.554866272 | 0.014707419 | 0.23718378 | 7.411415939 | 6.774625434 |
| SLC12A9 | 0.636045447 | 1.554063492 | 0.016116752 | 0.241534226 | 3.585910277 | 2.94986483 |
| PFKFB4 | 0.633480542 | 1.551303045 | 0.017148111 | 0.249432159 | 3.2867967 | 2.653316158 |
| RGS2 | 0.62850839 | 1.545965785 | 0.033755655 | 0.3125719 | 6.039450498 | 5.410942107 |
| BRI3 | 0.627186062 | 1.544549451 | 0.029364525 | 0.299226141 | 4.661758634 | 4.034572572 |
| HSPA1A | 0.626595852 | 1.543917701 | 0.006050557 | 0.184509368 | 5.514209858 | 4.887614006 |
| LRFN1 | 0.623416128 | 1.540518628 | 0.000609746 | 0.109965644 | 3.08250231 | 2.459086182 |
| HPCAL1 | 0.620333364 | 1.537230348 | 0.017733169 | 0.253547252 | 3.62495802 | 3.004624656 |
| FRAT1 | 0.616867252 | 1.533541546 | 0.018577552 | 0.258402138 | 5.004167235 | 4.387299984 |
| CCDC88B | 0.616185045 | 1.532816552 | 0.002268768 | 0.147692828 | 4.389224951 | 3.773039906 |
| SNX11 | 0.615793787 | 1.53240091 | 0.006679879 | 0.187584476 | 3.53719 | 2.921396213 |
| PLOD1 | 0.615409777 | 1.531993076 | 0.016438848 | 0.242282487 | 2.924846275 | 2.309436498 |
| GPR108 | 0.614845722 | 1.531394225 | 0.001136438 | 0.127294316 | 4.014600192 | 3.39975447 |
| TTYH3 | 0.61455975 | 1.531090702 | 0.015691942 | 0.240016619 | 3.709064752 | 3.094505002 |
| TCIRG1 | 0.614450867 | 1.530975151 | 0.028501281 | 0.297044096 | 5.963924245 | 5.349473379 |
| AATK | 0.613566896 | 1.530037376 | 0.040069118 | 0.335239934 | 3.904234354 | 3.290667458 |
| AP002807.1 | 0.613022839 | 1.52946049 | 0.032960374 | 0.309507647 | 1.462067904 | 0.849045065 |
| BCL6 | 0.611973082 | 1.528348004 | 0.01250465 | 0.223934 | 4.293105943 | 3.681132861 |
| ADAMTSL4 | 0.611561101 | 1.527911626 | 0.014374251 | 0.235755583 | 2.361628898 | 1.750067797 |
| DOK1 | 0.611370104 | 1.527709361 | 0.003017546 | 0.156159451 | 3.664481077 | 3.053110973 |
| PLAUR | 0.611334952 | 1.527672138 | 0.0007289 | 0.11797581 | 3.537212681 | 2.92587773 |
| AL022328.4 | 0.610964077 | 1.527279468 | 0.002794334 | 0.156159451 | 1.286946992 | 0.675982915 |
| PLP2 | 0.610951131 | 1.527265764 | 0.006210742 | 0.185530272 | 6.45321692 | 5.842265788 |
| NFAM1 | 0.607201736 | 1.523301732 | 0.000916575 | 0.123482026 | 6.084861293 | 5.477659557 |
| ALDH3B1 | 0.606661876 | 1.522731815 | 0.001932885 | 0.143432071 | 2.902856211 | 2.296194335 |
| DNTTIP1 | 0.605512328 | 1.521518977 | 0.020376191 | 0.265188383 | 3.695400206 | 3.089887878 |
| SH3BP5L | 0.604984458 | 1.520962368 | 0.001689441 | 0.143432071 | 3.225277543 | 2.620293085 |
| JOSD2 | 0.603127732 | 1.519006172 | 0.031981228 | 0.307503181 | 2.950042107 | 2.346914376 |
| PYCARD | 0.602384529 | 1.51822386 | 0.035033278 | 0.319535082 | 5.10813151 | 4.505746981 |
| ITGB2 | 0.601860986 | 1.517673007 | 3.64484E-05 | 0.072473812 | 7.360024986 | 6.758164 |
| ANXA11 | 0.599789225 | 1.51549514 | 0.006421634 | 0.186909523 | 4.91096242 | 4.311173195 |
| NINJ1 | 0.597163223 | 1.512739135 | 0.0025163 | 0.15128476 | 5.88668922 | 5.289525997 |
| PPP1R18 | 0.588314383 | 1.503489073 | 0.005842149 | 0.18372809 | 6.42383572 | 5.835521337 |
| FAM160A2 | 0.587652533 | 1.502799492 | 0.000754146 | 0.11988222 | 2.414891193 | 1.82723866 |
| KIAA2013 | 0.586414406 | 1.501510336 | 0.040923418 | 0.337338383 | 4.778375281 | 4.191960875 |
| SLC37A2 | 0.586183767 | 1.501270313 | 0.002837753 | 0.156159451 | 2.010659462 | 1.424475695 |
| ULK1 | 0.586011846 | 1.501091423 | 0.024382048 | 0.278282326 | 3.719320973 | 3.133309127 |
| ADAM19 | 0.584982085 | 1.500020363 | 0.026991392 | 0.288794919 | 2.823698716 | 2.23871663 |
| CAMKK2 | 0.584981486 | 1.500019739 | 0.01907941 | 0.260898472 | 2.759732367 | 2.174750881 |
| RGS19 | 0.584539436 | 1.499560195 | 0.004409587 | 0.171415457 | 6.389482295 | 5.80494286 |
| VASP | 0.582160202 | 1.49708922 | 0.000929375 | 0.123482026 | 6.836330916 | 6.254170714 |
| LAMTOR4 | 0.580453817 | 1.495319546 | 0.024831274 | 0.279850937 | 5.047754652 | 4.467300835 |
| INAFM1 | 0.577774942 | 1.492545531 | 0.00988112 | 0.207123899 | 3.341486258 | 2.763711316 |
| C1RL | 0.577753819 | 1.492523678 | 0.009448533 | 0.206584049 | 2.446488864 | 1.868735045 |
| ADAM8 | 0.577440957 | 1.492200045 | 0.030314465 | 0.303212775 | 6.47475678 | 5.897315823 |
| REM2 | 0.574491205 | 1.489152191 | 0.00171677 | 0.143432071 | 2.020198495 | 1.445707291 |
| OGFR | 0.572068591 | 1.486653664 | 0.006905788 | 0.188985286 | 4.599692582 | 4.027623991 |
| USB1 | 0.571650168 | 1.486222554 | 0.0061997 | 0.185530272 | 2.949199143 | 2.377548974 |
| ARHGEF11 | 0.570583139 | 1.485123738 | 0.000287287 | 0.079919692 | 2.401154361 | 1.830571221 |
| SLC15A3 | 0.569621266 | 1.484133907 | 0.007570513 | 0.195055265 | 3.951105211 | 3.381483945 |
| CFP | 0.569462339 | 1.483970425 | 0.039868802 | 0.334192159 | 5.349069169 | 4.77960683 |
| ST3GAL4 | 0.56878996 | 1.483278969 | 0.035752656 | 0.321160585 | 2.463145279 | 1.894355319 |
| CD82 | 0.565472648 | 1.479872257 | 0.002900982 | 0.156159451 | 4.282705625 | 3.717232978 |
| CTSL | 0.56237519 | 1.476698389 | 0.003132051 | 0.159322955 | 1.350192258 | 0.787817068 |
| MT1F | 0.560833564 | 1.47512127 | 0.011190297 | 0.216556577 | 1.412382904 | 0.85154934 |
| SIGLEC9 | 0.558073778 | 1.472302152 | 0.010996864 | 0.214645011 | 3.576094077 | 3.018020299 |
| B3GALT4 | 0.557602182 | 1.471820957 | 0.003031427 | 0.156159451 | 2.926645131 | 2.369042949 |
| LSP1 | 0.557540294 | 1.471757821 | 0.008471676 | 0.199237174 | 6.912390278 | 6.354849984 |
| TFEB | 0.552185038 | 1.466304811 | 0.017848706 | 0.253547252 | 3.811610245 | 3.259425207 |
| FKBP1C | 0.552145438 | 1.466264563 | 0.003881276 | 0.164726463 | 1.699627523 | 1.147482085 |
| GPAT3 | 0.550277052 | 1.464366883 | 0.022923216 | 0.273175997 | 1.909759981 | 1.359482929 |
| RAB32 | 0.549973507 | 1.464058811 | 0.048443363 | 0.36663797 | 4.639923471 | 4.089949964 |
| ZDHHC18 | 0.549081421 | 1.463153794 | 0.035672117 | 0.320760796 | 6.130727769 | 5.581646348 |
| CTSA | 0.547863378 | 1.461918999 | 0.009024897 | 0.203391487 | 4.439282025 | 3.891418647 |
| GRK6 | 0.547649177 | 1.46170196 | 0.02020369 | 0.264653675 | 5.427605018 | 4.879955841 |
| RAB24 | 0.546725822 | 1.46076674 | 0.037463521 | 0.327925526 | 2.76640133 | 2.219675507 |
| ALOX5 | 0.545533019 | 1.459559494 | 0.005365645 | 0.179567556 | 5.308436656 | 4.762903637 |
| TPD52L2 | 0.542501976 | 1.456496238 | 0.003177771 | 0.160203084 | 3.830210604 | 3.287708628 |
| TSC22D4 | 0.542107198 | 1.456097739 | 0.001949593 | 0.143432071 | 5.519950472 | 4.977843274 |
| ARAP1 | 0.541747028 | 1.455734268 | 0.002364064 | 0.150320716 | 5.404431012 | 4.862683984 |
| TUBA4A | 0.540694913 | 1.454673031 | 0.033855837 | 0.312964339 | 4.839884942 | 4.299190029 |
| HIST1H2BK | 0.537614421 | 1.451570277 | 0.04279173 | 0.34484178 | 5.996948751 | 5.45933433 |
| CORO1A | 0.537125227 | 1.451078157 | 0.017989247 | 0.253547252 | 8.046642776 | 7.509517549 |
| SBNO2 | 0.535906927 | 1.449853295 | 0.020969612 | 0.266811776 | 5.134499519 | 4.598592592 |
| CSF3R | 0.535523277 | 1.449467792 | 0.009604812 | 0.206584049 | 7.488615079 | 6.953091802 |
| TOR4A | 0.534608871 | 1.448549385 | 0.036555295 | 0.323626082 | 3.39825399 | 2.863645119 |
| MICAL1 | 0.534072008 | 1.448010444 | 0.004673947 | 0.175385196 | 3.508599349 | 2.974527341 |
| G6PD | 0.532623164 | 1.446556991 | 0.013872405 | 0.233612476 | 4.897645075 | 4.365021911 |
| ITGAX | 0.532511762 | 1.446445296 | 0.003976735 | 0.165183141 | 4.536737185 | 4.004225422 |
| UNC13D | 0.532447891 | 1.44638126 | 0.00924916 | 0.205252455 | 4.693493901 | 4.16104601 |
| HS1BP3 | 0.532313405 | 1.446246437 | 0.004211323 | 0.170405458 | 2.055140582 | 1.522827176 |
| ARHGAP4 | 0.531663495 | 1.445595074 | 0.028596584 | 0.297044096 | 4.871533989 | 4.339870494 |
| ORAI3 | 0.529049937 | 1.442978633 | 0.002918391 | 0.156159451 | 2.321297855 | 1.792247918 |
| NAGK | 0.528297665 | 1.442226409 | 0.046544562 | 0.359669874 | 3.387955331 | 2.859657666 |
| ANKRD13D | 0.527670818 | 1.441599902 | 0.045823917 | 0.35628329 | 3.510842491 | 2.983171672 |
| IL17RA | 0.527381518 | 1.441310851 | 0.010413195 | 0.20972458 | 4.9073921 | 4.380010581 |
| GNA15 | 0.526999016 | 1.440928766 | 0.046725071 | 0.360438977 | 2.939367355 | 2.412368339 |
| CD37 | 0.526968818 | 1.440898606 | 0.031658275 | 0.306661554 | 6.318661681 | 5.791692863 |
| ADAP1 | 0.52416145 | 1.438097459 | 0.045244606 | 0.354237011 | 2.432282809 | 1.908121359 |
| LRRC4 | 0.523685117 | 1.437622723 | 0.018324001 | 0.257064018 | 2.343021422 | 1.819336304 |
| C5AR2 | 0.519870699 | 1.433826736 | 0.001158261 | 0.127294316 | 2.899115599 | 2.3792449 |
| PLIN4 | 0.519563717 | 1.433521674 | 0.000835182 | 0.121855199 | 1.170319606 | 0.650755888 |
| SEMA4B | 0.519561568 | 1.433519538 | 0.001024205 | 0.124883735 | 3.362016945 | 2.842455377 |
| CYBC1 | 0.518517006 | 1.432481995 | 0.008388537 | 0.199237174 | 4.169834588 | 3.651317582 |
| NCKAP5L | 0.518399374 | 1.432365201 | 0.009198064 | 0.204801572 | 2.048564759 | 1.530165385 |
| LIMD2 | 0.517625222 | 1.431596798 | 0.007583734 | 0.195055265 | 6.118331579 | 5.600706357 |
| MTX1 | 0.515332291 | 1.429323313 | 0.01073662 | 0.212867508 | 2.001179079 | 1.485846788 |
| SIPA1 | 0.514815708 | 1.42881161 | 0.006866505 | 0.188985286 | 5.695771152 | 5.180955444 |
| LRP10 | 0.513301319 | 1.427312581 | 0.009700307 | 0.206584049 | 6.118470275 | 5.605168956 |
| RARA | 0.51190628 | 1.425933083 | 0.000876065 | 0.121855199 | 4.716010713 | 4.204104433 |
| NADK | 0.51165535 | 1.42568509 | 0.021427187 | 0.267202595 | 5.551847463 | 5.040192113 |
| PSAP | 0.51151968 | 1.425551026 | 0.003047353 | 0.156159451 | 9.033615184 | 8.522095505 |
| PISD | 0.511285617 | 1.425319764 | 0.02928024 | 0.298913642 | 3.828772409 | 3.317486792 |
| NEK6 | 0.5092658 | 1.423325668 | 0.009666472 | 0.206584049 | 2.047302736 | 1.538036936 |
| ZDHHC12 | 0.508702058 | 1.422769604 | 0.027717676 | 0.294091476 | 3.432082647 | 2.923380588 |
| PSENEN | 0.507652889 | 1.421735301 | 0.023274706 | 0.27442574 | 2.433478785 | 1.925825896 |
| TWF2 | 0.506140047 | 1.420245219 | 0.01559136 | 0.239956533 | 5.198833459 | 4.692693412 |
| ANXA9 | 0.504641051 | 1.418770316 | 0.002208303 | 0.147692828 | 2.23762739 | 1.732986339 |
| MGAT1 | 0.504029943 | 1.41816947 | 0.005473934 | 0.18182448 | 4.359024644 | 3.8549947 |
| ANPEP | 0.503205784 | 1.417359552 | 0.020139125 | 0.264653675 | 5.819603756 | 5.316397972 |
| RELT | 0.502890746 | 1.417050081 | 0.001291998 | 0.133695019 | 2.397431592 | 1.894540846 |
| TNNT1 | 0.501733751 | 1.415914108 | 0.023811832 | 0.276006413 | 1.072825481 | 0.57109173 |
| ABCD1 | 0.500779127 | 1.414977514 | 0.022101086 | 0.269512151 | 3.022405979 | 2.521626852 |
| BAK1 | 0.499139198 | 1.413370006 | 0.009412439 | 0.206584049 | 3.638543709 | 3.139404511 |
| IL4R | 0.498087986 | 1.412340537 | 0.0117008 | 0.219835934 | 4.001931532 | 3.503843546 |
| DGAT2 | 0.498065194 | 1.412318224 | 0.022443413 | 0.271508961 | 3.6208903 | 3.122825106 |
| ZBTB47 | 0.497762762 | 1.412022191 | 0.000172744 | 0.072473812 | 1.34239226 | 0.844629498 |
| CHMP2A | 0.496915349 | 1.411193039 | 0.021713329 | 0.267717531 | 5.107833749 | 4.6109184 |
| ARMC7 | 0.495977811 | 1.410276269 | 0.013663762 | 0.232754301 | 2.437554538 | 1.941576727 |
| MXD3 | 0.495618137 | 1.409924722 | 0.005861467 | 0.18372809 | 1.929649127 | 1.43403099 |
| FGR | 0.495215755 | 1.409531535 | 0.003223195 | 0.160203084 | 6.573982732 | 6.078766977 |
| ZNF687 | 0.4948226 | 1.40914747 | 0.000474406 | 0.097359587 | 2.975058543 | 2.480235943 |
| CEACAM3 | 0.494643532 | 1.408972577 | 0.049533995 | 0.369300499 | 3.549299455 | 3.054655923 |
| TMEM184B | 0.492587526 | 1.406966059 | 0.00183129 | 0.143432071 | 2.480854067 | 1.988266541 |
| PPM1M | 0.490719772 | 1.405145739 | 0.014552687 | 0.235970895 | 4.113194011 | 3.622474239 |
| FADD | 0.490200046 | 1.404639632 | 0.001908031 | 0.143432071 | 2.448581162 | 1.958381116 |
| DNAJC5 | 0.490017145 | 1.404461567 | 0.003036398 | 0.156159451 | 4.251780541 | 3.761763395 |
| MAP3K11 | 0.489368496 | 1.40383025 | 0.014024713 | 0.233797738 | 3.865906285 | 3.376537788 |
| NUP214 | 0.48803239 | 1.402530739 | 0.009629496 | 0.206584049 | 2.593481224 | 2.105448834 |
| MAP1S | 0.487767403 | 1.402273152 | 0.021420575 | 0.267202595 | 3.36102526 | 2.873257857 |
| PILRA | 0.48759265 | 1.402103306 | 0.006942057 | 0.188985286 | 5.738739433 | 5.251146783 |
| FGD3 | 0.487435634 | 1.401950716 | 0.000227956 | 0.072473812 | 4.971167534 | 4.483731901 |
| FCER1G | 0.48668425 | 1.401220742 | 0.021160926 | 0.266811776 | 7.187524306 | 6.700840056 |
| SLC9A8 | 0.4857381 | 1.400302094 | 0.008139235 | 0.198750611 | 2.036751554 | 1.551013453 |
| C11orf68 | 0.48478919 | 1.399381369 | 0.011454025 | 0.218404293 | 4.400421712 | 3.915632522 |
| RIN3 | 0.484429521 | 1.399032542 | 0.008941595 | 0.20318822 | 4.450578778 | 3.966149257 |
| ARAP3 | 0.484343494 | 1.398949122 | 0.023708111 | 0.276006413 | 3.297155474 | 2.812811979 |
| ATP6V0D1 | 0.48290655 | 1.397556442 | 0.023161282 | 0.273813724 | 4.301275889 | 3.818369339 |
| FMNL1 | 0.482894954 | 1.39754521 | 0.000108243 | 0.072473812 | 5.709311052 | 5.226416098 |
| ARHGAP9 | 0.482590757 | 1.397250563 | 0.00472069 | 0.175385196 | 5.164695409 | 4.682104652 |
| PRR13P5 | 0.481570636 | 1.396262925 | 0.036759849 | 0.32463906 | 3.07399567 | 2.592425034 |
| CMTM7 | 0.481491122 | 1.396185973 | 0.005926129 | 0.184509368 | 2.983311102 | 2.501819979 |
| AC099489.1 | 0.480604025 | 1.395327737 | 0.022579877 | 0.271508961 | 1.583880241 | 1.103276216 |
| MYBPC3 | 0.479878385 | 1.394626099 | 0.025436189 | 0.282335356 | 0.97236422 | 0.492485835 |
| CNPY3 | 0.478315617 | 1.393116218 | 0.018742358 | 0.258525185 | 6.370138234 | 5.891822617 |
| ATP6AP1 | 0.47736992 | 1.39220332 | 0.030081896 | 0.302244961 | 4.160486606 | 3.683116686 |
| COQ8B | 0.476866239 | 1.391717351 | 0.015508668 | 0.239956533 | 2.077345859 | 1.60047962 |
| MAST3 | 0.476590779 | 1.39145165 | 0.016323813 | 0.241740542 | 3.961998924 | 3.485408145 |
| GPANK1 | 0.47650872 | 1.391372508 | 0.002962234 | 0.156159451 | 2.2834043 | 1.80689558 |
| HMOX1 | 0.476025353 | 1.390906414 | 0.048534711 | 0.36663797 | 4.417328688 | 3.941303335 |
| ARID5A | 0.475593363 | 1.390489994 | 0.004304437 | 0.170726769 | 3.870830466 | 3.395237103 |
| GMIP | 0.475243886 | 1.390153204 | 0.003052319 | 0.156159451 | 5.407242187 | 4.931998301 |
| CCND3 | 0.475004843 | 1.389922886 | 0.0046488 | 0.175385196 | 5.337290059 | 4.862285217 |
| HEIH | 0.474865736 | 1.389788874 | 0.007957921 | 0.196236597 | 2.569416387 | 2.094550651 |
| MINDY1 | 0.474731701 | 1.38965976 | 0.010950107 | 0.214559226 | 3.930328784 | 3.455597083 |
| CTDSP1 | 0.474633275 | 1.389564956 | 0.009501678 | 0.206584049 | 5.284582186 | 4.809948911 |
| LILRB2 | 0.47461299 | 1.389545418 | 0.003335233 | 0.160203084 | 4.666317912 | 4.191704922 |
| TMUB2 | 0.47419323 | 1.38914118 | 0.010102783 | 0.207852 | 3.305430786 | 2.831237556 |
| DBNL | 0.472279683 | 1.387299887 | 0.000277786 | 0.079919692 | 3.621555185 | 3.149275503 |
| CTSZ | 0.471861697 | 1.386898008 | 0.002143781 | 0.147692828 | 6.018495107 | 5.54663341 |
| DAPK3 | 0.469603448 | 1.384728797 | 0.006525687 | 0.187584476 | 2.850155265 | 2.380551817 |
| CHMP1A | 0.468048285 | 1.383236923 | 0.003860593 | 0.164726463 | 4.292534205 | 3.82448592 |
| EHBP1L1 | 0.46754023 | 1.382749893 | 0.002736325 | 0.156159451 | 4.787823042 | 4.320282812 |
| LINGO3 | 0.467390279 | 1.38260618 | 0.025994825 | 0.285824708 | 3.049946464 | 2.582556185 |
| ARID3A | 0.466604751 | 1.381853574 | 0.013208589 | 0.230103444 | 1.997018445 | 1.530413694 |
| AC108134.3 | 0.464820457 | 1.380145584 | 0.01210359 | 0.221502022 | 1.258252098 | 0.793431641 |
| ZNF385A | 0.464704009 | 1.380034189 | 0.031328682 | 0.305129022 | 4.223825306 | 3.759121298 |
| RHBDD2 | 0.463283378 | 1.37867593 | 0.011599569 | 0.219234314 | 3.693676313 | 3.230392935 |
| KLHL21 | 0.461794474 | 1.377253829 | 0.048175965 | 0.36663797 | 3.448883753 | 2.987089279 |
| RAB43 | 0.460565832 | 1.376081418 | 0.019604965 | 0.2628972 | 1.554728857 | 1.094163025 |
| TMEM38A | 0.459150752 | 1.374732339 | 0.000204803 | 0.072473812 | 0.886077651 | 0.426926899 |
| MRPL28 | 0.45897525 | 1.374565114 | 0.025066963 | 0.281315602 | 3.090844165 | 2.631868915 |
| CLDN9 | 0.457102499 | 1.37278196 | 0.022985124 | 0.273182337 | 1.195551799 | 0.7384493 |
| TPP1 | 0.456250006 | 1.371971019 | 0.000187624 | 0.072473812 | 4.128023641 | 3.671773635 |
| FLII | 0.456183181 | 1.371907471 | 0.000361251 | 0.083896316 | 4.396041981 | 3.9398588 |
| AC215522.2 | 0.455467034 | 1.371226631 | 0.037963665 | 0.328109269 | 1.876799489 | 1.421332455 |
| EPN1 | 0.454781441 | 1.370575156 | 0.031278272 | 0.305129022 | 2.522871248 | 2.068089807 |
| ACAA1 | 0.453973068 | 1.369807408 | 0.005955647 | 0.184509368 | 2.448581904 | 1.994608836 |
| CNN2 | 0.4537669 | 1.36961167 | 0.026971829 | 0.288794919 | 6.847491267 | 6.393724367 |
| RNASEK | 0.451619735 | 1.367574791 | 0.03249412 | 0.308093548 | 1.561921871 | 1.110302136 |
| ZNF865 | 0.450776689 | 1.366775876 | 0.042484214 | 0.343558805 | 3.265607926 | 2.814831236 |
| KDM6B | 0.449282533 | 1.36536108 | 0.009160651 | 0.204801572 | 4.993551187 | 4.544268654 |
| C15orf39 | 0.448637731 | 1.364750978 | 0.005350662 | 0.179567556 | 5.510135321 | 5.06149759 |
| RNF181 | 0.44778355 | 1.363943185 | 0.047128838 | 0.361673203 | 3.766008416 | 3.318224866 |
| POR | 0.447391929 | 1.363572991 | 0.017717175 | 0.253547252 | 3.062586432 | 2.615194503 |
| PXN | 0.447128836 | 1.36332435 | 0.000386402 | 0.083896316 | 4.995487042 | 4.548358206 |
| ZNF581 | 0.44712659 | 1.363322228 | 0.040308863 | 0.336368149 | 3.207060701 | 2.759934111 |
| TECPR2 | 0.446458471 | 1.362691012 | 0.006072362 | 0.184509368 | 2.635042699 | 2.188584228 |
| TNFRSF1A | 0.446455934 | 1.362688616 | 0.029768546 | 0.300929118 | 6.046194687 | 5.599738753 |
| KCNQ1 | 0.44463067 | 1.360965665 | 0.038896057 | 0.33076938 | 2.887204571 | 2.442573901 |
| AL627309.7 | 0.44442835 | 1.360774819 | 0.030921291 | 0.304135878 | 4.750351416 | 4.305923066 |
| STRN4 | 0.444208499 | 1.360567468 | 0.017770673 | 0.253547252 | 2.987292434 | 2.543083935 |
| WAS | 0.444111937 | 1.360476406 | 0.001744943 | 0.143432071 | 6.464613358 | 6.020501421 |
| DEDD2 | 0.443971321 | 1.36034381 | 0.04862902 | 0.36663797 | 5.32527348 | 4.881302159 |
| MVP | 0.443706407 | 1.360094041 | 0.00880606 | 0.202040062 | 5.195888982 | 4.752182575 |
| ARID3B | 0.443633166 | 1.360024995 | 0.000551579 | 0.104471476 | 2.320479879 | 1.876846713 |
| PLCB2 | 0.442527801 | 1.358983369 | 0.000644589 | 0.109965644 | 3.879007343 | 3.436479542 |
| SH2D3C | 0.441670347 | 1.358175909 | 9.92283E-05 | 0.072473812 | 4.338414848 | 3.896744501 |
| XPO6 | 0.438940699 | 1.355608604 | 0.033778271 | 0.3125719 | 5.434529505 | 4.995588806 |
| CLIC1 | 0.438845956 | 1.355519584 | 0.006670218 | 0.187584476 | 6.931016442 | 6.492170486 |
| TADA3 | 0.438683491 | 1.355366943 | 0.007845747 | 0.195055265 | 3.867315883 | 3.428632392 |
| UBOX5 | 0.437743758 | 1.354484381 | 0.001048065 | 0.124883735 | 1.403925951 | 0.966182193 |
| PRR13 | 0.437728166 | 1.354469743 | 0.035387132 | 0.320466173 | 4.847714366 | 4.4099862 |
| NCF1B | 0.437501813 | 1.354257249 | 0.003643069 | 0.164622344 | 5.725438766 | 5.287936953 |
| RAC2 | 0.437238989 | 1.354010559 | 0.012138282 | 0.221502022 | 7.483845382 | 7.046606393 |
| TBC1D10B | 0.434913046 | 1.351829354 | 0.026768966 | 0.288794919 | 3.515048623 | 3.080135577 |
| ACAP1 | 0.434638623 | 1.351572239 | 0.044594958 | 0.351313553 | 3.891657046 | 3.457018423 |
| MGST2 | 0.434531365 | 1.351471759 | 0.01484782 | 0.23815368 | 1.307976859 | 0.873445494 |
| RTL8A | 0.4331201 | 1.350150376 | 0.000216562 | 0.072473812 | 2.073434987 | 1.640314887 |
| NOP10 | 0.432357824 | 1.349437187 | 0.026123163 | 0.286037389 | 5.74150792 | 5.309150096 |
| NR1H2 | 0.43187107 | 1.348981974 | 0.032639394 | 0.308118652 | 4.337332803 | 3.905461732 |
| ZNF408 | 0.431762361 | 1.34888033 | 0.033264202 | 0.311039072 | 1.773182897 | 1.341420536 |
| ATG2A | 0.430706645 | 1.347893625 | 0.04913105 | 0.367533281 | 3.482437234 | 3.051730589 |
| JPT1 | 0.430023007 | 1.347255062 | 0.049777964 | 0.369300499 | 4.124619379 | 3.694596372 |
| MMP25-AS1 | 0.429448696 | 1.34671885 | 0.019653569 | 0.2628972 | 1.943558579 | 1.514109883 |
| LYPLA2 | 0.428342613 | 1.345686746 | 0.041258642 | 0.338895232 | 4.013480159 | 3.585137546 |
| MOB3A | 0.425728279 | 1.34325041 | 0.003198927 | 0.160203084 | 6.466892276 | 6.041163998 |
| PRKCD | 0.422396922 | 1.340152261 | 0.021344147 | 0.267202595 | 4.680490844 | 4.258093922 |
| ABHD8 | 0.4210473 | 1.338899153 | 0.043594708 | 0.346768856 | 2.076146301 | 1.655099001 |
| GGT1 | 0.420646048 | 1.338526822 | 0.021491469 | 0.267202595 | 1.765443409 | 1.34479736 |
| GSN | 0.42054099 | 1.338429353 | 0.007863966 | 0.195055265 | 3.542212606 | 3.121671616 |
| ZNF319 | 0.420417997 | 1.338315254 | 0.001995013 | 0.145570558 | 2.830255931 | 2.409837933 |
| TNFRSF1B | 0.42028741 | 1.338194119 | 0.006128491 | 0.185530272 | 7.003390183 | 6.583102773 |
| MYO1F | 0.420275366 | 1.338182948 | 0.009691729 | 0.206584049 | 6.339583759 | 5.919308393 |
| AL359762.1 | 0.418649061 | 1.336675306 | 0.037953354 | 0.328109269 | 1.641191439 | 1.222542378 |
| DNM2 | 0.418167358 | 1.336229077 | 0.002158218 | 0.147692828 | 3.372939754 | 2.954772396 |
| PTPN6 | 0.417437528 | 1.335553276 | 0.007161278 | 0.191444492 | 5.692610259 | 5.275172732 |
| CHPF2 | 0.417195479 | 1.335329222 | 0.028927854 | 0.297706073 | 3.602199663 | 3.185004184 |
| VPS18 | 0.416892308 | 1.335048643 | 0.044677761 | 0.351380628 | 3.026682627 | 2.609790319 |
| TYK2 | 0.416265993 | 1.334469186 | 0.049542921 | 0.369300499 | 4.229065766 | 3.812799773 |
| CARD9 | 0.415692881 | 1.333939172 | 0.026311629 | 0.286690482 | 1.448518703 | 1.032825823 |
| CCM2 | 0.415382774 | 1.333652473 | 0.00583758 | 0.18372809 | 3.160012475 | 2.744629701 |
| PLEKHM1 | 0.414449545 | 1.332790059 | 0.00294596 | 0.156159451 | 2.869754867 | 2.455305322 |
| EHD1 | 0.41189542 | 1.330432595 | 0.011668149 | 0.219835934 | 5.194328676 | 4.782433256 |
| SH3BP2 | 0.410822916 | 1.329443915 | 0.012161997 | 0.221502022 | 3.120381722 | 2.709558807 |
| ARSA | 0.409542913 | 1.328264915 | 0.009888526 | 0.207123899 | 4.124247079 | 3.714704166 |
| PKN1 | 0.409070133 | 1.327829706 | 0.010810331 | 0.212867508 | 5.221358358 | 4.812288225 |
| STARD3 | 0.407743626 | 1.326609375 | 0.010362518 | 0.20972458 | 3.103482075 | 2.695738449 |
| MGRN1 | 0.407454883 | 1.326343892 | 0.00120784 | 0.131124266 | 2.936328793 | 2.528873911 |
| WBP2 | 0.406881222 | 1.3258166 | 0.03973738 | 0.333719018 | 5.126956161 | 4.720074939 |
| LILRB4 | 0.406291168 | 1.32527446 | 0.003409638 | 0.160203084 | 1.70290157 | 1.296610402 |
| GPR132 | 0.405443485 | 1.324495999 | 0.000549557 | 0.104471476 | 2.186440398 | 1.780996912 |
| CXCR4 | 0.405239648 | 1.324308875 | 0.035073073 | 0.319570621 | 5.366653713 | 4.961414064 |
| DTX2 | 0.404448162 | 1.323582536 | 0.014198911 | 0.235755583 | 2.393591045 | 1.989142883 |
| PTK2B | 0.404422254 | 1.323558768 | 0.001903464 | 0.143432071 | 4.205345972 | 3.800923718 |
| CUEDC2 | 0.403899576 | 1.323079339 | 0.030441124 | 0.303405312 | 3.314617372 | 2.910717795 |
| MAPKAPK2 | 0.403115818 | 1.322360758 | 0.032517134 | 0.308093548 | 4.459896575 | 4.056780758 |
| RPS6KA1 | 0.402904711 | 1.322167274 | 0.00691874 | 0.188985286 | 4.946656877 | 4.543752166 |
| ITPRIP | 0.402011559 | 1.321348992 | 0.047086098 | 0.361656984 | 3.322656814 | 2.920645256 |
| PGAM1 | 0.400514027 | 1.319978131 | 0.001902124 | 0.143432071 | 5.004387731 | 4.603873704 |
| MBD6 | 0.399131829 | 1.318714109 | 0.020730229 | 0.265908505 | 3.342393825 | 2.943261996 |
| COTL1 | 0.398771909 | 1.318385161 | 0.006354796 | 0.186909523 | 6.688476676 | 6.289704766 |
| TRABD | 0.398151611 | 1.317818432 | 0.019298793 | 0.261630844 | 3.976615169 | 3.578463558 |
| SLC38A10 | 0.397451108 | 1.317178718 | 0.026821192 | 0.288794919 | 3.677803852 | 3.280352744 |
| SRA1 | 0.397292588 | 1.317033998 | 0.03582537 | 0.321165607 | 2.711811577 | 2.314518989 |
| PDK4 | 0.396952975 | 1.316724002 | 0.048340335 | 0.36663797 | 0.962636335 | 0.56568336 |
| SLC9A1 | 0.396138007 | 1.315980404 | 0.011361012 | 0.218404293 | 2.60381103 | 2.207673024 |
| CALCOCO1 | 0.395450649 | 1.315353568 | 0.001017329 | 0.124883735 | 2.682032802 | 2.286582153 |
| NIT1 | 0.395438488 | 1.31534248 | 0.000420235 | 0.089069751 | 1.711944705 | 1.316506217 |
| TNFRSF12A | 0.394692751 | 1.314662748 | 0.039050936 | 0.331393168 | 1.455354124 | 1.060661373 |
| ADGRE5 | 0.394425501 | 1.314419238 | 0.0441572 | 0.348790948 | 6.98651151 | 6.592086009 |
| CMTM3 | 0.394407579 | 1.31440291 | 0.036841673 | 0.325039216 | 3.782531318 | 3.388123739 |
| EFHD2 | 0.393584448 | 1.31365319 | 0.003558857 | 0.161961772 | 7.586448177 | 7.192863729 |
| CCDC97 | 0.39179923 | 1.312028656 | 0.009822172 | 0.207123899 | 3.56120126 | 3.16940203 |
| SLC26A6 | 0.391794848 | 1.312024671 | 0.003995248 | 0.165183141 | 1.038245651 | 0.646450803 |
| FBRS | 0.39073368 | 1.311059971 | 0.023710247 | 0.276006413 | 3.904601873 | 3.513868193 |
| LINC00921 | 0.390631108 | 1.310966762 | 0.023588388 | 0.276006413 | 1.042844762 | 0.652213654 |
| TMEM127 | 0.389724583 | 1.310143268 | 0.007695046 | 0.195055265 | 4.011226772 | 3.621502189 |
| GRK2 | 0.389359787 | 1.30981203 | 0.038469886 | 0.329762578 | 5.780815957 | 5.391456171 |
| MAPK13 | 0.389295604 | 1.30975376 | 0.015914937 | 0.240470911 | 2.027172506 | 1.637876902 |
| ZNF282 | 0.388592576 | 1.309115671 | 0.002640283 | 0.153619628 | 2.320589419 | 1.931996843 |
| RAB34 | 0.38807288 | 1.308644178 | 0.00996815 | 0.207400749 | 1.650460182 | 1.262387302 |
| CAPN1 | 0.387202551 | 1.307854956 | 0.032576532 | 0.308093548 | 4.171896634 | 3.784694083 |
| FOSL2 | 0.387121853 | 1.307781802 | 0.005540029 | 0.182656823 | 3.837509822 | 3.450387969 |
| BCKDK | 0.385636295 | 1.306435859 | 0.034364353 | 0.315372652 | 2.970796778 | 2.585160483 |
| GALNS | 0.385149042 | 1.3059947 | 0.004956996 | 0.176488433 | 1.710086914 | 1.324937872 |
| B3GNT2 | 0.384868866 | 1.305741096 | 0.02477204 | 0.279850937 | 1.85955796 | 1.474689094 |
| THAP8 | 0.384056723 | 1.305006256 | 0.007696827 | 0.195055265 | 1.017331571 | 0.633274848 |
| LASP1 | 0.380831224 | 1.302091853 | 0.00089799 | 0.122983148 | 5.09647118 | 4.715639957 |
| PARVG | 0.380178048 | 1.301502468 | 0.037182109 | 0.32642518 | 2.896672584 | 2.516494537 |
| CSTB | 0.379413153 | 1.300812615 | 0.047069813 | 0.361656984 | 3.184327814 | 2.80491466 |
| RAB1B | 0.379412823 | 1.300812317 | 0.039096612 | 0.331464804 | 5.400997738 | 5.021584914 |
| ARPC4 | 0.377541274 | 1.29912592 | 0.013475266 | 0.232357708 | 4.516308894 | 4.13876762 |
| STK40 | 0.377294614 | 1.298903826 | 0.004974868 | 0.176488433 | 4.860530831 | 4.483236217 |
| CANT1 | 0.377211833 | 1.298829298 | 0.009271715 | 0.205252455 | 4.153939827 | 3.776727994 |
| ITGA5 | 0.377111467 | 1.298738943 | 0.01546884 | 0.239956533 | 4.243013215 | 3.865901749 |
| ST3GAL2 | 0.376751509 | 1.298414943 | 0.008149177 | 0.198750611 | 2.921978337 | 2.545226828 |
| COLGALT1 | 0.376233567 | 1.297948883 | 0.037155899 | 0.32642518 | 3.168208258 | 2.791974691 |
| STYXL1 | 0.373409582 | 1.295410714 | 0.04325064 | 0.346141435 | 1.798037927 | 1.424628345 |
| FHOD1 | 0.372920257 | 1.294971419 | 0.021652945 | 0.2673433 | 2.280077946 | 1.907157689 |
| PLXND1 | 0.372303461 | 1.294417898 | 0.020554634 | 0.265908505 | 2.503650257 | 2.131346797 |
| C9orf139 | 0.369730366 | 1.292111318 | 0.003914108 | 0.164726463 | 1.152639195 | 0.782908828 |
| SLC25A44 | 0.367193559 | 1.289841291 | 0.020156632 | 0.264653675 | 2.500923215 | 2.133729655 |
| THEMIS2 | 0.366964401 | 1.289636428 | 0.023808889 | 0.276006413 | 5.206286296 | 4.839321896 |
| TMEM234 | 0.366160499 | 1.288918014 | 0.016374921 | 0.241740542 | 0.988672513 | 0.622512014 |
| SMCO4 | 0.365065856 | 1.28794042 | 0.019413285 | 0.26206474 | 2.317472708 | 1.952406852 |
| TMEM150B | 0.364701775 | 1.287615434 | 0.013467443 | 0.232357708 | 1.457641417 | 1.092939642 |
| STAT6 | 0.364611084 | 1.287534494 | 0.004792351 | 0.175561749 | 4.865801777 | 4.501190693 |
| HCK | 0.364314443 | 1.287269784 | 0.026908882 | 0.288794919 | 6.150777221 | 5.786462778 |
| MFSD5 | 0.363923082 | 1.286920633 | 0.02626996 | 0.286690482 | 2.954355972 | 2.59043289 |
| SLCO3A1 | 0.362206737 | 1.285390519 | 0.006575096 | 0.187584476 | 2.180031655 | 1.817824918 |
| SELPLG | 0.361513661 | 1.284773162 | 0.035520303 | 0.320691417 | 7.345977725 | 6.984464064 |
| VPS37B | 0.361349841 | 1.284627282 | 0.029949606 | 0.301818145 | 2.905922784 | 2.544572943 |
| SLC12A4 | 0.360201787 | 1.28360542 | 0.002051936 | 0.147309127 | 1.244814271 | 0.884612484 |
| FCHSD1 | 0.359611162 | 1.283080033 | 0.011285735 | 0.217929743 | 2.058027015 | 1.698415852 |
| CD2BP2 | 0.359287114 | 1.282791869 | 0.017957379 | 0.253547252 | 3.556509568 | 3.197222453 |
| INSL3 | 0.357532902 | 1.281233035 | 0.010082865 | 0.207852 | 0.992038871 | 0.634505969 |
| PLA2G15 | 0.35691934 | 1.280688257 | 0.008233073 | 0.199237174 | 1.254785842 | 0.897866501 |
| ATP6V0A1 | 0.356836914 | 1.280615089 | 0.031749943 | 0.306881642 | 1.737380664 | 1.380543751 |
| ELL | 0.356398194 | 1.280225717 | 0.005404966 | 0.180206031 | 3.10441155 | 2.748013356 |
| EEPD1 | 0.356392487 | 1.280220652 | 0.022080249 | 0.269512151 | 1.842644074 | 1.486251588 |
| ALAS1 | 0.355656757 | 1.279567946 | 0.004605868 | 0.175385196 | 2.577715151 | 2.222058394 |
| RNF167 | 0.354378582 | 1.278434797 | 0.048332295 | 0.36663797 | 4.629351015 | 4.274972433 |
| TMC4 | 0.353384994 | 1.277554639 | 0.027208133 | 0.289897629 | 0.913277148 | 0.559892154 |
| TCTA | 0.351042805 | 1.275482235 | 0.000281488 | 0.079919692 | 2.198214065 | 1.84717126 |
| RFT1 | 0.350902004 | 1.27535776 | 0.003463631 | 0.160859338 | 1.009346122 | 0.658444117 |
| FAM53C | 0.350164158 | 1.274705663 | 0.018506411 | 0.257828865 | 3.267837367 | 2.917673209 |
| SELENON | 0.349660019 | 1.274260304 | 0.034089016 | 0.313492174 | 2.677327379 | 2.32766736 |
| AC004151.1 | 0.349044742 | 1.273716977 | 0.047334706 | 0.362618121 | 6.165195536 | 5.816150793 |
| MYPOP | 0.348578682 | 1.273305571 | 0.036105279 | 0.322226786 | 1.726102581 | 1.377523899 |
| CSK | 0.348128484 | 1.272908294 | 0.028222651 | 0.295553315 | 5.906183012 | 5.558054528 |
| RTL8C | 0.346175531 | 1.271186344 | 0.040608673 | 0.336368149 | 3.382191266 | 3.036015735 |
| ZNF516 | 0.345870441 | 1.270917551 | 0.028248968 | 0.295553315 | 2.198588831 | 1.85271839 |
| MRTFA | 0.344684811 | 1.26987352 | 0.004673031 | 0.175385196 | 3.731025838 | 3.386341027 |
| MAPRE3 | 0.344578637 | 1.269780068 | 0.03053701 | 0.303405312 | 1.054973667 | 0.71039503 |
| MARK2 | 0.34457106 | 1.269773399 | 0.013016933 | 0.228789675 | 3.581176366 | 3.236605306 |
| UBA1 | 0.344116306 | 1.269373215 | 0.025219451 | 0.281315602 | 4.708100961 | 4.363984654 |
| MYH9 | 0.34369012 | 1.268998285 | 0.045462726 | 0.355153841 | 6.930929741 | 6.587239621 |
| ZBTB48 | 0.342388073 | 1.267853518 | 0.032467069 | 0.308093548 | 2.045312203 | 1.702924131 |
| GDI1 | 0.342212786 | 1.267699483 | 0.030896177 | 0.304135878 | 3.556883358 | 3.214670572 |
| NPEPL1 | 0.340262991 | 1.265987351 | 0.007199391 | 0.191444492 | 1.089222525 | 0.748959535 |
| LINC01503 | 0.339745851 | 1.265533635 | 0.04229855 | 0.343558805 | 0.916334183 | 0.576588332 |
| PAQR4 | 0.338329729 | 1.26429202 | 0.010453849 | 0.209954512 | 0.844983951 | 0.506654222 |
| NAGA | 0.337406292 | 1.263483034 | 0.03813782 | 0.329121424 | 3.260440096 | 2.923033804 |
| NOMO2 | 0.335595211 | 1.261897921 | 0.016265893 | 0.241740542 | 1.359862706 | 1.024267494 |
| CDC42EP4 | 0.335216034 | 1.261566306 | 0.016653508 | 0.244636183 | 1.198795963 | 0.863579929 |
| ZNF688 | 0.33479551 | 1.261198632 | 0.013985017 | 0.23374783 | 1.981023992 | 1.646228482 |
| SNHG30 | 0.333669346 | 1.260214528 | 0.045549306 | 0.355372412 | 0.922280929 | 0.588611583 |
| ZMAT5 | 0.333652882 | 1.260200146 | 0.014261058 | 0.235755583 | 1.264755729 | 0.931102847 |
| WWP2 | 0.333570479 | 1.260128169 | 0.030538212 | 0.303405312 | 3.224543798 | 2.890973319 |
| PRR14 | 0.33247395 | 1.259170765 | 0.043355167 | 0.346141435 | 3.01242166 | 2.67994771 |
| MNT | 0.331208636 | 1.258066895 | 0.000853731 | 0.121855199 | 1.558192771 | 1.226984135 |
| TRIOBP | 0.330493335 | 1.257443288 | 0.001242967 | 0.133312004 | 1.912016079 | 1.581522745 |
| ARHGDIA | 0.329170063 | 1.256290462 | 0.04751663 | 0.363084155 | 4.942013217 | 4.612843154 |
| CYHR1 | 0.327253067 | 1.254622262 | 0.04069518 | 0.336368149 | 2.212452833 | 1.885199766 |
| CSRP1 | 0.327175322 | 1.254554654 | 0.007257605 | 0.191712758 | 1.487004416 | 1.159829094 |
| F12 | 0.326955934 | 1.25436389 | 0.008448887 | 0.199237174 | 0.593056071 | 0.266100138 |
| FOLR2 | 0.32665956 | 1.254106232 | 0.037728458 | 0.328109269 | 1.081448526 | 0.754788966 |
| CDC42SE1 | 0.326460668 | 1.253933351 | 0.024307323 | 0.278282326 | 5.009272092 | 4.682811423 |
| PDLIM2 | 0.326445892 | 1.253920508 | 0.012644517 | 0.225122973 | 2.232164669 | 1.905718777 |
| ARHGEF1 | 0.325712775 | 1.25328348 | 0.039149611 | 0.331598325 | 4.714166204 | 4.388453429 |
| TLN1 | 0.325681128 | 1.253255989 | 0.025539284 | 0.283126653 | 6.42272253 | 6.097041401 |
| NEU1 | 0.325400502 | 1.253012235 | 0.019195931 | 0.261617729 | 2.311079531 | 1.985679029 |
| BEST1 | 0.324578665 | 1.252298655 | 0.044248271 | 0.349200449 | 1.277869327 | 0.953290661 |
| DGCR2 | 0.324247019 | 1.252010811 | 0.007170785 | 0.191444492 | 4.14899582 | 3.824748801 |
| ZNF775 | 0.323930013 | 1.251735734 | 0.028854154 | 0.297635777 | 1.172026285 | 0.848096273 |
| KDM4B | 0.32256319 | 1.250550389 | 0.013747909 | 0.232754301 | 2.80924119 | 2.486678 |
| CAMTA2 | 0.321232291 | 1.249397277 | 0.013882386 | 0.233612476 | 2.82909968 | 2.507867389 |
| AL118506.1 | 0.320492351 | 1.248756641 | 0.0489577 | 0.36730648 | 0.90133109 | 0.58083874 |
| ITPKC | 0.318681588 | 1.247190279 | 0.011994581 | 0.221502022 | 1.522229927 | 1.203548338 |
| RNF166 | 0.318450032 | 1.246990118 | 0.042240462 | 0.343558805 | 4.249468815 | 3.931018783 |
| GLTP | 0.318126415 | 1.246710432 | 0.021089616 | 0.266811776 | 1.868546663 | 1.550420248 |
| KIF1C | 0.318030233 | 1.246627318 | 0.042847122 | 0.344868968 | 1.784658277 | 1.466628044 |
| MUL1 | 0.317607688 | 1.246262252 | 0.007774331 | 0.195055265 | 2.677867276 | 2.360259588 |
| USP19 | 0.316430948 | 1.245246148 | 0.031113148 | 0.305094908 | 3.161312947 | 2.844881999 |
| THBS3 | 0.315606978 | 1.24453515 | 0.002191851 | 0.147692828 | 0.889869885 | 0.574262907 |
| GLB1 | 0.315555549 | 1.244490786 | 0.005017642 | 0.176488433 | 2.596427499 | 2.280871949 |
| CRACR2B | 0.31512521 | 1.244119625 | 0.030689455 | 0.304135878 | 0.591149253 | 0.276024043 |
| KATNB1 | 0.314360735 | 1.243460548 | 0.024892144 | 0.280138895 | 2.189409118 | 1.875048383 |
| FBP1 | 0.313925522 | 1.243085494 | 0.041942121 | 0.342862115 | 2.496710816 | 2.182785294 |
| ATXN7L3 | 0.313619716 | 1.242822027 | 0.001018654 | 0.124883735 | 3.208994901 | 2.895375185 |
| BAIAP2-DT | 0.313424879 | 1.242654194 | 0.006671272 | 0.187584476 | 1.402458883 | 1.089034004 |
| IQCN | 0.3116428 | 1.241120162 | 0.00016888 | 0.072473812 | 0.612520859 | 0.300878059 |
| SFXN5 | 0.308780076 | 1.238659862 | 0.010801294 | 0.212867508 | 0.833781216 | 0.52500114 |
| MMP14 | 0.308753006 | 1.238636621 | 0.037581814 | 0.328109269 | 0.726728522 | 0.417975516 |
| INPPL1 | 0.308427622 | 1.238357291 | 0.040424548 | 0.336368149 | 3.127439874 | 2.819012252 |
| HM13 | 0.308364445 | 1.238303064 | 0.031812374 | 0.306986966 | 2.677454427 | 2.369089983 |
| C10orf105 | 0.307566718 | 1.237618543 | 0.0056969 | 0.183035808 | 0.688631149 | 0.381064431 |
| KIF3C | 0.307476239 | 1.237540928 | 0.015446255 | 0.239956533 | 1.251144768 | 0.943668529 |
| DNASE1L1 | 0.306146545 | 1.236400845 | 0.013520702 | 0.232357708 | 1.153965533 | 0.847818988 |
| ACSS2 | 0.30367032 | 1.234280521 | 0.022094805 | 0.269512151 | 1.875652195 | 1.571981876 |
| NT5C3B | 0.303099108 | 1.233791924 | 0.03698864 | 0.325368449 | 1.018042569 | 0.714943461 |
| TICAM1 | 0.299994964 | 1.231140116 | 0.02483512 | 0.279850937 | 2.648299991 | 2.348305027 |
| MSRA | 0.299450007 | 1.230675159 | 0.006605342 | 0.187584476 | 1.305489423 | 1.006039416 |
| SERTAD3 | 0.29564089 | 1.227430119 | 0.040873793 | 0.337338383 | 2.561086704 | 2.265445814 |
| UPF1 | 0.295559891 | 1.227361208 | 0.03124108 | 0.305129022 | 3.683863374 | 3.388303483 |
| RELA | 0.294577208 | 1.226525483 | 0.009518172 | 0.206584049 | 3.50983373 | 3.215256522 |
| REEP5 | 0.294547279 | 1.226500038 | 0.043187551 | 0.346044624 | 2.658136375 | 2.363589096 |
| DOK4 | 0.293864927 | 1.225920077 | 0.006424884 | 0.186909523 | 0.710892946 | 0.417028019 |
| SIL1 | 0.292024735 | 1.224357383 | 0.006416626 | 0.186909523 | 1.229684956 | 0.937660221 |
| LAMB2 | 0.291534618 | 1.223941512 | 0.002272963 | 0.147692828 | 0.548320407 | 0.256785789 |
| KDM5C | 0.29138913 | 1.22381809 | 0.012503786 | 0.223934 | 2.724763921 | 2.433374791 |
| NSFL1C | 0.291017695 | 1.223503047 | 0.010220837 | 0.208205702 | 2.368424363 | 2.077406668 |
| MYO9B | 0.289986246 | 1.222628622 | 0.003846615 | 0.164726463 | 3.915751993 | 3.625765747 |
| TRIP6 | 0.289520255 | 1.222233776 | 0.014338327 | 0.235755583 | 0.888113268 | 0.598593013 |
| SLC44A2 | 0.288888284 | 1.221698495 | 0.018507374 | 0.257828865 | 5.820340813 | 5.531452529 |
| CFL1 | 0.288766158 | 1.221595081 | 0.033377628 | 0.311039072 | 6.038200149 | 5.749433991 |
| ORAI2 | 0.286822954 | 1.21995079 | 0.006625946 | 0.187584476 | 3.021305882 | 2.734482928 |
| LRSAM1 | 0.28672706 | 1.219869704 | 0.013030371 | 0.228789675 | 1.640049537 | 1.353322477 |
| TRIM62 | 0.285786525 | 1.219074695 | 0.023270527 | 0.27442574 | 1.584912995 | 1.29912647 |
| PSD4 | 0.285125097 | 1.218515917 | 0.001774176 | 0.143432071 | 3.563747205 | 3.278622108 |
| EPHB6 | 0.284391431 | 1.217896413 | 0.019236154 | 0.261617729 | 1.896277111 | 1.611885679 |
| LAMP1 | 0.284377915 | 1.217885003 | 0.000209354 | 0.072473812 | 4.483525097 | 4.199147182 |
| IL2RG | 0.282445892 | 1.216255132 | 0.038329864 | 0.329355642 | 4.487607806 | 4.205161914 |
| PHF12 | 0.280116612 | 1.214293031 | 0.019969208 | 0.264653675 | 1.899416591 | 1.61929998 |
| ATOX1 | 0.279325529 | 1.213627372 | 0.049782139 | 0.369300499 | 1.087692122 | 0.808366593 |
| ABR | 0.278938956 | 1.213302221 | 0.008947403 | 0.20318822 | 2.304835296 | 2.02589634 |
| GNAI2 | 0.277745492 | 1.212298937 | 0.032602126 | 0.308093548 | 5.800181241 | 5.522435749 |
| TNFSF12 | 0.277305482 | 1.211929252 | 0.038833228 | 0.33076938 | 3.315807899 | 3.038502417 |
| NCSTN | 0.273549151 | 1.208777868 | 0.007720522 | 0.195055265 | 3.621479212 | 3.347930061 |
| GIT1 | 0.272249059 | 1.207689062 | 0.039287604 | 0.332135089 | 2.524696418 | 2.252447359 |
| MYO18A | 0.271834644 | 1.207342202 | 0.01874703 | 0.258525185 | 1.810846731 | 1.539012087 |
| FAM219A | 0.271172339 | 1.206788069 | 0.005713425 | 0.183035808 | 1.387590292 | 1.116417953 |
| MTMR14 | 0.269545915 | 1.205428362 | 0.003016772 | 0.156159451 | 2.83384236 | 2.564296445 |
| ARHGAP1 | 0.268479938 | 1.204538025 | 0.016986867 | 0.247527479 | 3.299927105 | 3.031447168 |
| RIPK3 | 0.265656589 | 1.202183054 | 0.043012318 | 0.345573698 | 1.773419094 | 1.507762505 |
| PLIN5 | 0.263755689 | 1.200600096 | 0.047510293 | 0.363084155 | 0.991678538 | 0.727922849 |
| DHRS7B | 0.263081976 | 1.200039569 | 0.005523837 | 0.182656823 | 0.731271261 | 0.468189284 |
| PDHA1 | -0.263122161 | 0.833282645 | 0.006041665 | 0.184509368 | 0.800433859 | 1.06355602 |
| BUB3 | -0.264145553 | 0.832691757 | 0.019309309 | 0.261630844 | 0.822016589 | 1.086162142 |
| FBXW4 | -0.265025709 | 0.832183905 | 0.036945786 | 0.325312943 | 1.516658435 | 1.781684143 |
| MCM3 | -0.266670219 | 0.831235849 | 0.012674065 | 0.225198649 | 1.291609556 | 1.558279775 |
| LY9 | -0.266776517 | 0.831174606 | 0.007831906 | 0.195055265 | 0.877793101 | 1.144569618 |
| XRCC6 | -0.267001953 | 0.831044736 | 0.03581609 | 0.321165607 | 3.479349473 | 3.746351426 |
| ZNF831 | -0.269559447 | 0.829572832 | 0.014979376 | 0.238544546 | 0.520665451 | 0.790224898 |
| TAF9 | -0.270131758 | 0.82924381 | 0.030451126 | 0.303405312 | 0.618132714 | 0.888264471 |
| PAFAH1B2 | -0.271209981 | 0.828624292 | 0.028500457 | 0.297044096 | 0.982338185 | 1.253548165 |
| ST13P4 | -0.271299107 | 0.828573103 | 0.026717983 | 0.288794919 | 0.233172423 | 0.50447153 |
| CSTF2 | -0.271491619 | 0.828462546 | 0.042194685 | 0.343558805 | 0.468103513 | 0.739595132 |
| MLH1 | -0.272687287 | 0.827776222 | 0.011935981 | 0.221362705 | 0.612294803 | 0.88498209 |
| UBE2G2 | -0.27325056 | 0.827453096 | 0.008237354 | 0.199237174 | 1.06265528 | 1.33590584 |
| QSOX2 | -0.27390658 | 0.827076923 | 0.037752265 | 0.328109269 | 0.755277235 | 1.029183815 |
| ZBTB40 | -0.274386667 | 0.826801742 | 0.015928192 | 0.240470911 | 0.54537512 | 0.819761787 |
| FLVCR1 | -0.275090992 | 0.826398195 | 0.000722569 | 0.11797581 | 0.151869078 | 0.42696007 |
| NCOA5 | -0.275146349 | 0.826366486 | 0.022425456 | 0.271508961 | 1.523189089 | 1.798335438 |
| DNAJC19 | -0.275279099 | 0.826290451 | 0.008565797 | 0.199237174 | 0.485398494 | 0.760677593 |
| GPA33 | -0.275765253 | 0.826012058 | 0.009202497 | 0.204801572 | 0.556164873 | 0.831930126 |
| ATP8B2 | -0.277037447 | 0.825283987 | 0.031604873 | 0.306477754 | 1.536659386 | 1.813696833 |
| NUP188 | -0.277520792 | 0.825007539 | 0.002516333 | 0.15128476 | 1.099632138 | 1.37715293 |
| SLC35E1 | -0.278332366 | 0.82454357 | 0.008133306 | 0.198750611 | 1.17091182 | 1.449244186 |
| TFG | -0.278701824 | 0.82433244 | 0.018029141 | 0.253547252 | 0.91103797 | 1.189739794 |
| SEPHS1 | -0.279186463 | 0.824055572 | 0.015219333 | 0.238946208 | 0.680522493 | 0.959708956 |
| AC005332.4 | -0.279252367 | 0.824017929 | 0.029971606 | 0.301818145 | 0.462150882 | 0.741403249 |
| PTPN4 | -0.279295992 | 0.823993012 | 0.03084483 | 0.304135878 | 0.360397333 | 0.639693325 |
| KANSL2 | -0.279341215 | 0.823967184 | 0.012351666 | 0.2225944 | 0.752858725 | 1.03219994 |
| CYB5B | -0.279528555 | 0.823860195 | 0.004008038 | 0.165183141 | 0.902951828 | 1.182480384 |
| C11orf58 | -0.280102302 | 0.823532618 | 0.032150027 | 0.308072704 | 0.914378638 | 1.19448094 |
| PSMG2 | -0.280872966 | 0.823092818 | 0.020706384 | 0.265908505 | 1.200759751 | 1.481632717 |
| MBTPS1 | -0.281391329 | 0.822797132 | 0.00213492 | 0.147692828 | 0.923396616 | 1.204787945 |
| DPH2 | -0.282256697 | 0.822303744 | 0.014950213 | 0.238506799 | 0.983262921 | 1.265519618 |
| UBIAD1 | -0.282873644 | 0.821952174 | 0.048668471 | 0.36663797 | 0.981787573 | 1.264661217 |
| PABPC4 | -0.2834032 | 0.821650523 | 0.022822913 | 0.273034118 | 1.712873733 | 1.996276933 |
| ZCCHC17 | -0.283549367 | 0.821567282 | 0.010131394 | 0.207852 | 1.190159586 | 1.473708953 |
| TSPOAP1 | -0.284294762 | 0.821142913 | 0.042036709 | 0.343312647 | 0.469203838 | 0.753498599 |
| CDYL | -0.287929287 | 0.819076844 | 0.010815452 | 0.212867508 | 0.532719512 | 0.820648799 |
| UXS1 | -0.289670413 | 0.818088931 | 0.012060152 | 0.221502022 | 0.494291574 | 0.783961987 |
| NIPSNAP1 | -0.290209046 | 0.817783553 | 0.015418804 | 0.239956533 | 1.319435074 | 1.60964412 |
| PAFAH2 | -0.292761069 | 0.816338232 | 0.009012948 | 0.203391487 | 0.799042548 | 1.091803617 |
| MRPS27 | -0.294298334 | 0.815468846 | 0.01108004 | 0.214890011 | 0.544905215 | 0.839203549 |
| AC005261.2 | -0.295060755 | 0.815038009 | 0.035497021 | 0.320691417 | 0.41444198 | 0.709502736 |
| ANXA7 | -0.295737926 | 0.814655537 | 0.037884756 | 0.328109269 | 2.209381364 | 2.50511929 |
| CCDC117 | -0.29676589 | 0.814075276 | 0.018760646 | 0.258525185 | 0.616324471 | 0.913090361 |
| ELAVL1 | -0.300028899 | 0.812236126 | 0.024746969 | 0.279850937 | 1.13746989 | 1.437498788 |
| MEAF6 | -0.302630013 | 0.810773021 | 0.020017376 | 0.264653675 | 1.185026767 | 1.48765678 |
| SRSF3 | -0.3035916 | 0.810232803 | 0.037442148 | 0.327925526 | 1.837756068 | 2.141347668 |
| NSUN2 | -0.305233761 | 0.809311072 | 0.016310006 | 0.241740542 | 0.901063285 | 1.206297046 |
| FAM122B | -0.305321892 | 0.809261635 | 0.014483943 | 0.235755583 | 1.133633853 | 1.438955744 |
| UROS | -0.305956293 | 0.808905854 | 0.019448672 | 0.26206474 | 0.611040976 | 0.916997269 |
| PPP1R8 | -0.308222153 | 0.807636405 | 0.032706063 | 0.308420945 | 1.271582046 | 1.579804198 |
| EIF2D | -0.308827921 | 0.80729736 | 0.009378392 | 0.206584049 | 0.747428008 | 1.056255929 |
| MRPS15 | -0.308869011 | 0.807274368 | 0.04977653 | 0.369300499 | 0.881270616 | 1.190139627 |
| VDAC1 | -0.309206207 | 0.807085708 | 0.00017405 | 0.072473812 | 1.843140291 | 2.152346498 |
| STK39 | -0.309601575 | 0.806864558 | 0.014474646 | 0.235755583 | 0.371653671 | 0.681255246 |
| CPSF6 | -0.309892581 | 0.806701822 | 0.013602475 | 0.232754301 | 0.667721969 | 0.97761455 |
| RALA | -0.310252161 | 0.806500783 | 0.030791814 | 0.304135878 | 0.693437444 | 1.003689605 |
| H2AFV | -0.312031957 | 0.805506448 | 0.014104299 | 0.234684988 | 1.68995124 | 2.001983197 |
| YWHAE | -0.312068568 | 0.805486007 | 0.046347888 | 0.358772956 | 1.998741488 | 2.310810057 |
| BEX2 | -0.312748314 | 0.80510658 | 0.03657284 | 0.323626082 | 0.614588675 | 0.927336989 |
| RPL7L1 | -0.315167672 | 0.803757571 | 0.033113822 | 0.310293939 | 0.606017869 | 0.921185542 |
| HSPA9 | -0.315358039 | 0.803651521 | 0.044682416 | 0.351380628 | 0.950452087 | 1.265810125 |
| PRSS23 | -0.315408838 | 0.803623224 | 0.015626879 | 0.239956533 | 0.305900502 | 0.62130934 |
| CLK1 | -0.317694293 | 0.802351167 | 0.0377563 | 0.328109269 | 1.583780424 | 1.901474716 |
| UBE2Q2 | -0.318537238 | 0.801882502 | 0.044138616 | 0.348790948 | 0.622235401 | 0.940772639 |
| OCIAD1 | -0.319005507 | 0.80162227 | 0.018668542 | 0.258525185 | 0.973367156 | 1.292372663 |
| GID8 | -0.319185893 | 0.801522046 | 0.001339469 | 0.133695019 | 1.636134104 | 1.955319996 |
| ESD | -0.320566376 | 0.800755454 | 0.023781889 | 0.276006413 | 1.123240753 | 1.443807129 |
| RCC2 | -0.320764018 | 0.800645762 | 0.015960315 | 0.240470911 | 2.291498694 | 2.612262713 |
| GID4 | -0.32245711 | 0.799706706 | 0.00602991 | 0.184509368 | 0.470797252 | 0.793254362 |
| PREP | -0.324316025 | 0.798676946 | 8.76383E-05 | 0.072473812 | 1.13513311 | 1.459449135 |
| MGAT4A | -0.324821334 | 0.798397255 | 0.029170625 | 0.298595146 | 0.565421437 | 0.890242771 |
| ILF3 | -0.326226428 | 0.797620045 | 0.021895682 | 0.268821513 | 2.042773166 | 2.368999594 |
| TOMM20 | -0.326455083 | 0.797493639 | 0.034026557 | 0.313492174 | 1.941615417 | 2.268070501 |
| ERBB2 | -0.327829102 | 0.79673447 | 0.038262278 | 0.329121424 | 0.358110776 | 0.685939879 |
| UQCRC2 | -0.328074682 | 0.796598859 | 0.024376409 | 0.278282326 | 1.491280026 | 1.819354708 |
| MTMR1 | -0.330886021 | 0.795048061 | 0.028094073 | 0.295553315 | 0.897264005 | 1.228150026 |
| NCAPD2 | -0.331851821 | 0.794516 | 0.025154831 | 0.281315602 | 1.368479843 | 1.700331664 |
| TC2N | -0.332107693 | 0.7943751 | 0.02501282 | 0.28114157 | 0.561429443 | 0.893537136 |
| PDIA4 | -0.333131207 | 0.793811734 | 0.003264406 | 0.160203084 | 1.748858377 | 2.081989583 |
| ALDH18A1 | -0.333443756 | 0.793639779 | 0.03241777 | 0.308093548 | 0.876872742 | 1.210316497 |
| PAM | -0.334116957 | 0.793269531 | 0.03564601 | 0.320760796 | 0.314481888 | 0.648598845 |
| TMED10 | -0.33493738 | 0.792818548 | 0.003983468 | 0.165183141 | 2.163769178 | 2.498706558 |
| CCND2 | -0.335671015 | 0.792415489 | 0.015136535 | 0.238910339 | 0.632947767 | 0.968618783 |
| INTS14 | -0.337558866 | 0.791379245 | 0.002881952 | 0.156159451 | 0.491321119 | 0.828879985 |
| ERMP1 | -0.338851751 | 0.79067036 | 0.002750653 | 0.156159451 | 0.350860098 | 0.689711849 |
| FCRL3 | -0.341281926 | 0.789339622 | 0.028240661 | 0.295553315 | 0.325916231 | 0.667198157 |
| PDAP1 | -0.341581048 | 0.789175981 | 0.028792525 | 0.297635777 | 2.620793784 | 2.962374831 |
| MRFAP1L1 | -0.341670371 | 0.789127121 | 0.025184922 | 0.281315602 | 2.206253845 | 2.547924217 |
| ARHGEF12 | -0.343006011 | 0.788396889 | 0.020612599 | 0.265908505 | 0.336598421 | 0.679604431 |
| ANAPC16 | -0.343018669 | 0.788389972 | 0.004748802 | 0.175385196 | 1.996466132 | 2.339484801 |
| CACYBP | -0.345079724 | 0.787264471 | 0.008711604 | 0.201072176 | 0.605166182 | 0.950245906 |
| TP53RK | -0.345167187 | 0.787216744 | 0.006769768 | 0.188985286 | 1.066200738 | 1.411367925 |
| RMDN3 | -0.346830867 | 0.786309469 | 5.52803E-06 | 0.024605259 | 0.649778612 | 0.996609479 |
| TIGIT | -0.34721033 | 0.786102678 | 0.002422402 | 0.15111156 | 0.505472354 | 0.852682684 |
| ILF2 | -0.353485634 | 0.782690786 | 0.014323293 | 0.235755583 | 1.995815545 | 2.349301179 |
| DNAJB9 | -0.355945119 | 0.781357603 | 0.019120318 | 0.261056858 | 0.557064444 | 0.913009563 |
| P2RY11 | -0.357764433 | 0.780372891 | 0.000313482 | 0.082076848 | 0.500660995 | 0.858425428 |
| PTGES3 | -0.35812608 | 0.780177296 | 0.039498005 | 0.33296519 | 2.654337724 | 3.012463804 |
| SERBP1 | -0.358510977 | 0.77996918 | 0.049524706 | 0.369300499 | 1.491056679 | 1.849567657 |
| SDE2 | -0.359375983 | 0.779501669 | 0.025933178 | 0.2857143 | 0.989592803 | 1.348968786 |
| AHSA1 | -0.361434405 | 0.778390278 | 0.012801747 | 0.226980487 | 1.925032918 | 2.286467322 |
| TMEM64 | -0.3623825 | 0.777878911 | 0.024363898 | 0.278282326 | 0.459797684 | 0.822180184 |
| TCP1 | -0.367135454 | 0.775320409 | 0.001776257 | 0.143432071 | 1.533686778 | 1.900822232 |
| TXK | -0.368334614 | 0.774676234 | 0.027899919 | 0.294970404 | 0.496511648 | 0.864846262 |
| HOPX | -0.374473602 | 0.771386818 | 0.024383309 | 0.278282326 | 0.576329079 | 0.950802682 |
| FAM168B | -0.376816996 | 0.770134859 | 0.003233355 | 0.160203084 | 1.636147071 | 2.012964067 |
| CHMP7 | -0.376848741 | 0.770117913 | 0.006572009 | 0.187584476 | 2.051881284 | 2.428730024 |
| SERBP1P1 | -0.380091147 | 0.768389044 | 0.043858235 | 0.347354096 | 0.463514225 | 0.843605372 |
| ALDH5A1 | -0.382053938 | 0.767344359 | 0.015064164 | 0.238910339 | 0.358808321 | 0.740862259 |
| ZFAND1 | -0.382301021 | 0.767212951 | 0.005055555 | 0.176488433 | 0.457851523 | 0.840152544 |
| CD5 | -0.38616458 | 0.765161092 | 0.032241187 | 0.308093548 | 3.061524171 | 3.447688752 |
| PDIA6 | -0.386598905 | 0.764930774 | 0.003859666 | 0.164726463 | 1.559133763 | 1.945732669 |
| TMED2 | -0.388304819 | 0.764026816 | 0.009192365 | 0.204801572 | 1.830825713 | 2.219130533 |
| SFPQ | -0.388868286 | 0.763728472 | 0.043959249 | 0.347844655 | 1.883814841 | 2.272683127 |
| CHI3L2 | -0.389142999 | 0.763583059 | 0.026007416 | 0.285824708 | 0.38179733 | 0.77094033 |
| AMD1 | -0.391486735 | 0.762343585 | 0.017786724 | 0.253547252 | 1.468389474 | 1.859876208 |
| EIF3M | -0.39182451 | 0.76216512 | 0.024766255 | 0.279850937 | 0.792883546 | 1.184708056 |
| ATIC | -0.392705026 | 0.761700092 | 0.023099314 | 0.273808382 | 1.103141661 | 1.495846687 |
| NSA2 | -0.395393608 | 0.760281922 | 0.002312993 | 0.14828481 | 0.603422395 | 0.998816003 |
| MEX3C | -0.399464419 | 0.75813968 | 0.008120221 | 0.198750611 | 0.590511829 | 0.989976248 |
| MRPL3 | -0.400000476 | 0.757858033 | 0.011449363 | 0.218404293 | 0.729671057 | 1.129671533 |
| EIF3B | -0.400034972 | 0.757839912 | 0.00371704 | 0.164726463 | 1.662315963 | 2.062350935 |
| PLCG1 | -0.400050533 | 0.757831738 | 0.017875748 | 0.253547252 | 1.538974412 | 1.939024945 |
| PCDH1 | -0.404030325 | 0.755744078 | 0.001106588 | 0.127294316 | 0.167918461 | 0.571948786 |
| RHOH | -0.40430146 | 0.75560206 | 0.009531709 | 0.206584049 | 0.794310456 | 1.198611916 |
| APBA2 | -0.404983546 | 0.755244906 | 0.001411687 | 0.135683908 | 1.045326831 | 1.450310377 |
| PWP1 | -0.40529429 | 0.755082251 | 0.039928763 | 0.334379914 | 0.865949979 | 1.271244269 |
| EIF4A2 | -0.40625201 | 0.754581162 | 0.035387357 | 0.320466173 | 2.242724766 | 2.648976777 |
| FASLG | -0.407233269 | 0.754068103 | 0.044946183 | 0.353142914 | 0.587196594 | 0.994429863 |
| LINC00861 | -0.416890895 | 0.749037112 | 0.032363105 | 0.308093548 | 0.907960504 | 1.324851398 |
| AC135050.6 | -0.416971078 | 0.748995482 | 0.02145231 | 0.267202595 | 1.518361948 | 1.935333026 |
| AC246787.1 | -0.421329597 | 0.74673611 | 0.031119543 | 0.305094908 | 0.322642842 | 0.743972439 |
| FUT11 | -0.425970391 | 0.744337904 | 0.021466818 | 0.267202595 | 0.712117067 | 1.138087458 |
| HMGB1 | -0.426778888 | 0.743920888 | 0.015993199 | 0.240470911 | 1.748793287 | 2.175572175 |
| NCR1 | -0.428116392 | 0.743231528 | 0.033437923 | 0.311039072 | 0.6801202 | 1.108236592 |
| SIGLEC17P | -0.429007068 | 0.742772821 | 0.002581469 | 0.152567726 | 0.421607694 | 0.850614762 |
| DCK | -0.433555699 | 0.740434643 | 0.020429608 | 0.265495428 | 1.300381954 | 1.733937653 |
| DNAJA4 | -0.434213892 | 0.740096915 | 0.048260292 | 0.36663797 | 0.578533724 | 1.012747616 |
| GFI1 | -0.434554963 | 0.739921968 | 0.004107432 | 0.166960554 | 0.63839398 | 1.072948944 |
| ZNF330 | -0.435311981 | 0.739533814 | 0.01186625 | 0.22052893 | 0.995833578 | 1.43114556 |
| PRPS1 | -0.435815332 | 0.739275839 | 0.016018788 | 0.240470911 | 1.38397284 | 1.819788172 |
| ATP5F1A | -0.436069543 | 0.739145585 | 0.011424078 | 0.218404293 | 2.031560373 | 2.467629917 |
| RPA1 | -0.436174305 | 0.739091914 | 0.01512937 | 0.238910339 | 1.68600508 | 2.122179385 |
| APEX1 | -0.436609727 | 0.738868881 | 0.021097743 | 0.266811776 | 2.870427266 | 3.307036993 |
| USP12 | -0.437989272 | 0.738162692 | 0.00457738 | 0.175385196 | 0.533809643 | 0.971798915 |
| NUDT21 | -0.438458451 | 0.737922673 | 0.013995461 | 0.23374783 | 0.993396455 | 1.431854906 |
| EIF3D | -0.439246852 | 0.737519525 | 0.005078706 | 0.176604062 | 2.991883619 | 3.431130471 |
| RASA3 | -0.439317765 | 0.737483274 | 0.045887609 | 0.356449824 | 2.90826221 | 3.347579975 |
| M6PR | -0.439803722 | 0.737234902 | 0.033913998 | 0.313176778 | 2.010933954 | 2.450737676 |
| MRPL45 | -0.441333179 | 0.736453745 | 0.028913574 | 0.297706073 | 1.104971658 | 1.546304837 |
| CCT4 | -0.443341742 | 0.735429146 | 0.038940273 | 0.33076938 | 1.91808471 | 2.361426452 |
| RCAN3 | -0.444170125 | 0.73500699 | 0.003916989 | 0.164726463 | 0.699667445 | 1.14383757 |
| SRSF1 | -0.446114285 | 0.734017169 | 0.017322558 | 0.249927735 | 1.175310282 | 1.621424567 |
| KLRD1 | -0.446196336 | 0.733975425 | 0.038655114 | 0.330555066 | 0.615670639 | 1.061866975 |
| ENDOD1 | -0.447564188 | 0.733279856 | 0.011506584 | 0.218404293 | 0.910678199 | 1.358242387 |
| STRAP | -0.447975083 | 0.733071039 | 0.026057737 | 0.286024632 | 1.733988252 | 2.181963335 |
| SUCLG2 | -0.448116422 | 0.732999225 | 0.004716569 | 0.175385196 | 0.547273323 | 0.995389745 |
| SRSF8 | -0.448768367 | 0.732668062 | 0.005827764 | 0.18372809 | 1.392559319 | 1.841327686 |
| C1QBP | -0.449754867 | 0.732167243 | 0.030797616 | 0.304135878 | 2.25027332 | 2.700028187 |
| AC022149.1 | -0.452062272 | 0.730997171 | 0.046687468 | 0.360438977 | 1.503682613 | 1.955744885 |
| FANCF | -0.45487588 | 0.729572937 | 0.015973039 | 0.240470911 | 0.72398836 | 1.17886424 |
| IL24 | -0.458835855 | 0.727573118 | 0.04371141 | 0.346768856 | 1.546793889 | 2.005629744 |
| NOL7 | -0.45942236 | 0.727277394 | 0.022828249 | 0.273034118 | 1.112296255 | 1.571718616 |
| RDH14 | -0.467688195 | 0.723122416 | 0.016365891 | 0.241740542 | 0.797363795 | 1.26505199 |
| ABLIM1 | -0.471070591 | 0.721429043 | 0.026667016 | 0.288794919 | 0.83435632 | 1.305426911 |
| AC115223.1 | -0.471185712 | 0.721371478 | 0.006536035 | 0.187584476 | 0.76491465 | 1.236100362 |
| ST6GAL1 | -0.473709999 | 0.720110397 | 0.035301956 | 0.320344562 | 1.947317469 | 2.421027468 |
| SET | -0.474991923 | 0.719470818 | 0.032803736 | 0.308688012 | 1.929526677 | 2.404518599 |
| SRSF7 | -0.476204141 | 0.71886654 | 0.003770249 | 0.164726463 | 1.357662004 | 1.833866145 |
| SRSF6 | -0.482184319 | 0.715892902 | 0.002177427 | 0.147692828 | 1.574136232 | 2.056320551 |
| ZBTB44 | -0.487288517 | 0.713364577 | 0.003565997 | 0.161961772 | 0.794208128 | 1.281496645 |
| UFC1 | -0.488244802 | 0.712891882 | 0.013113333 | 0.22976286 | 2.092474033 | 2.580718835 |
| PDZD4 | -0.489712914 | 0.7121668 | 0.031404626 | 0.305534404 | 1.728213762 | 2.217926676 |
| EIF3H | -0.491716342 | 0.711178521 | 0.001258448 | 0.133365517 | 1.593080695 | 2.084797037 |
| POLE3 | -0.492431579 | 0.710826031 | 0.002043198 | 0.147309127 | 1.4146421 | 1.90707368 |
| NLRC3 | -0.492977402 | 0.710557152 | 0.018419323 | 0.257407865 | 1.011695421 | 1.504672823 |
| GGTA1P | -0.497001405 | 0.708578008 | 0.036330436 | 0.322226786 | 0.548175631 | 1.045177036 |
| LBH | -0.499395952 | 0.707402905 | 0.007575663 | 0.195055265 | 2.950036424 | 3.449432376 |
| DENND2D | -0.500249886 | 0.706984315 | 0.000808395 | 0.121855199 | 1.79136267 | 2.291612556 |
| PDCD1 | -0.501033253 | 0.706600535 | 0.002683449 | 0.155117301 | 0.797055318 | 1.298088571 |
| RIOK3 | -0.50601513 | 0.704164729 | 0.029757061 | 0.300929118 | 1.398271429 | 1.904286559 |
| EID1 | -0.509214888 | 0.70260469 | 0.037880661 | 0.328109269 | 1.754214881 | 2.263429769 |
| CD40LG | -0.511687223 | 0.701401673 | 0.012825341 | 0.226980487 | 0.989888399 | 1.501575622 |
| SH2D1A | -0.516453523 | 0.699088243 | 0.004609169 | 0.175385196 | 0.367149351 | 0.883602874 |
| SPN | -0.519936452 | 0.697402552 | 0.047076178 | 0.361656984 | 3.375489517 | 3.89542597 |
| ADSS | -0.522375749 | 0.696224386 | 0.024210698 | 0.278282326 | 0.807766306 | 1.330142055 |
| HNRNPA1 | -0.524077874 | 0.695403448 | 0.008365103 | 0.199237174 | 3.96594393 | 4.490021804 |
| PRR5L | -0.529310584 | 0.692885762 | 0.021461981 | 0.267202595 | 1.061406888 | 1.590717472 |
| RPL36AL | -0.530995666 | 0.692076937 | 0.00892645 | 0.20318822 | 5.139707271 | 5.670702937 |
| GIMAP7 | -0.541593326 | 0.687011748 | 0.042933989 | 0.34525598 | 2.653913462 | 3.195506788 |
| ANP32B | -0.54623513 | 0.684804872 | 0.013664461 | 0.232754301 | 3.603579057 | 4.149814188 |
| TRGC1 | -0.547322975 | 0.684288698 | 0.010138066 | 0.207852 | 0.669249744 | 1.216572719 |
| CCT6A | -0.549988028 | 0.683025796 | 0.013608686 | 0.232754301 | 1.253925065 | 1.803913093 |
| YWHAQ | -0.555325789 | 0.68050337 | 0.020198515 | 0.264653675 | 2.72577248 | 3.281098269 |
| NCOA4 | -0.555360326 | 0.680487079 | 0.007427418 | 0.195040916 | 4.563915606 | 5.119275932 |
| SRP9 | -0.556951858 | 0.679736803 | 0.046961886 | 0.361656984 | 3.005822971 | 3.562774829 |
| HSPD1 | -0.556987361 | 0.679720076 | 0.007503917 | 0.195055265 | 1.110495563 | 1.667482925 |
| LEF1 | -0.557220258 | 0.679610356 | 0.011545444 | 0.218675626 | 1.364809497 | 1.922029756 |
| ACP1 | -0.560159974 | 0.678226954 | 0.008457745 | 0.199237174 | 1.289367505 | 1.84952748 |
| SLC25A38 | -0.560847201 | 0.677903958 | 0.04973125 | 0.369300499 | 2.140887102 | 2.701734303 |
| KLRG1 | -0.563758163 | 0.676537513 | 0.014632571 | 0.236404978 | 0.827142296 | 1.390900458 |
| TCF7 | -0.56663436 | 0.675190093 | 0.023789368 | 0.276006413 | 2.069937332 | 2.636571693 |
| RBM3 | -0.569800677 | 0.673709862 | 0.006486792 | 0.187578932 | 2.74586197 | 3.315662646 |
| CD247 | -0.572309547 | 0.672539287 | 0.042805006 | 0.34484178 | 3.042543567 | 3.614853114 |
| NAP1L4 | -0.576014826 | 0.670814219 | 0.000386012 | 0.083896316 | 1.326512278 | 1.902527104 |
| CD28 | -0.576757472 | 0.670468997 | 0.000786577 | 0.120725956 | 0.641369766 | 1.218127238 |
| CMPK1 | -0.577754728 | 0.670005699 | 0.028253861 | 0.295553315 | 2.162677474 | 2.740432202 |
| NFATC2 | -0.580454889 | 0.668752883 | 0.03182981 | 0.306986966 | 1.292178317 | 1.872633206 |
| RPL30 | -0.585352256 | 0.666486586 | 0.048609089 | 0.36663797 | 5.159331273 | 5.744683529 |
| NOG | -0.586471338 | 0.6659698 | 0.022012898 | 0.269512151 | 0.43559832 | 1.022069657 |
| HSP90AB1 | -0.590487176 | 0.664118606 | 0.017468466 | 0.251625061 | 3.508089884 | 4.09857706 |
| ST3GAL1 | -0.591856866 | 0.663488394 | 0.004078434 | 0.166573898 | 1.988770914 | 2.58062778 |
| EEF1A1P6 | -0.592555892 | 0.663166993 | 0.035257616 | 0.320344562 | 1.528699487 | 2.121255378 |
| SPCS2P4 | -0.601999601 | 0.658840158 | 0.036243695 | 0.322226786 | 2.058296764 | 2.660296365 |
| PNP | -0.602865994 | 0.658444618 | 0.047561313 | 0.363113899 | 1.894016773 | 2.496882767 |
| SSBP3 | -0.603524413 | 0.658144185 | 0.004767829 | 0.175385196 | 1.773387632 | 2.376912045 |
| SKAP1 | -0.604604819 | 0.657651498 | 0.020571957 | 0.265908505 | 1.509374826 | 2.113979645 |
| RPL23A | -0.604926694 | 0.657504788 | 0.031308252 | 0.305129022 | 5.134177879 | 5.739104573 |
| EIF3L | -0.609912474 | 0.655236453 | 0.014586189 | 0.236084105 | 2.544171017 | 3.154083491 |
| PTMA | -0.616086074 | 0.652438547 | 0.003317038 | 0.160203084 | 4.500133307 | 5.116219381 |
| NORAD | -0.617322178 | 0.651879776 | 0.010168445 | 0.207852 | 2.498165545 | 3.115487723 |
| SIRPG | -0.626553619 | 0.647721878 | 0.015973982 | 0.240470911 | 1.24386715 | 1.87042077 |
| EIF3E | -0.629902148 | 0.646220244 | 0.016284249 | 0.241740542 | 1.426213326 | 2.056115474 |
| TRAM1 | -0.630927775 | 0.645761003 | 0.022880639 | 0.273034118 | 1.917710106 | 2.548637881 |
| RPL14 | -0.641097331 | 0.64122504 | 0.005703271 | 0.183035808 | 3.066951317 | 3.708048648 |
| NPM1 | -0.641733381 | 0.640942401 | 0.004380676 | 0.171059357 | 1.990995142 | 2.632728524 |
| RPL22 | -0.642935739 | 0.640408455 | 0.012079904 | 0.221502022 | 1.999038714 | 2.641974453 |
| PA2G4 | -0.646795988 | 0.638697191 | 5.79496E-05 | 0.072473812 | 1.502233518 | 2.149029506 |
| RPS11 | -0.65389953 | 0.635560104 | 0.049911852 | 0.369676686 | 8.509731808 | 9.163631338 |
| RPL19 | -0.660484147 | 0.632665948 | 0.015876724 | 0.240470911 | 7.010065727 | 7.670549874 |
| PIP4K2A | -0.660826993 | 0.632515617 | 0.016910675 | 0.247190193 | 2.607940199 | 3.268767192 |
| RPL12P4 | -0.661981143 | 0.63200981 | 0.004675638 | 0.175385196 | 1.174958071 | 1.836939214 |
| PABPC1 | -0.666212515 | 0.630158864 | 0.032538223 | 0.308093548 | 4.483801537 | 5.150014052 |
| RPL11 | -0.669405892 | 0.628765562 | 0.025854429 | 0.285199658 | 5.610663624 | 6.280069516 |
| NACA | -0.671825516 | 0.627711909 | 0.000613629 | 0.109965644 | 2.391873542 | 3.063699058 |
| RPS25 | -0.676927429 | 0.625496003 | 0.046062094 | 0.357493249 | 6.195747652 | 6.872675081 |
| YY1AP1 | -0.68554629 | 0.621770343 | 0.035483905 | 0.320691417 | 2.02453547 | 2.710081761 |
| HIST1H2AG | -0.686108582 | 0.621528054 | 0.007866192 | 0.195055265 | 0.552803039 | 1.238911621 |
| RPL24 | -0.688016437 | 0.620706674 | 0.026278351 | 0.286690482 | 4.621084822 | 5.309101259 |
| ITM2A | -0.691110021 | 0.619377113 | 0.021008703 | 0.266811776 | 1.926350831 | 2.617460852 |
| CD2 | -0.694674492 | 0.617848705 | 0.040926247 | 0.337338383 | 2.886579096 | 3.581253588 |
| XCL2 | -0.695801147 | 0.617366392 | 0.033987394 | 0.313492174 | 1.065550544 | 1.76135169 |
| MT-TV | -0.696645121 | 0.617005339 | 0.015731917 | 0.240215315 | 4.695644208 | 5.392289329 |
| RPS20 | -0.698556139 | 0.616188585 | 0.04159641 | 0.340654317 | 5.611238299 | 6.309794439 |
| IGF2BP2 | -0.702408014 | 0.614545607 | 0.020360067 | 0.265188383 | 0.826403214 | 1.528811228 |
| TCEA1P2 | -0.724353498 | 0.605268217 | 0.006196058 | 0.185530272 | 0.82331819 | 1.547671688 |
| ARL4C | -0.73236633 | 0.601915833 | 0.022354361 | 0.271508961 | 2.682698182 | 3.415064511 |
| RPL7 | -0.734703037 | 0.600941709 | 0.049018209 | 0.36730648 | 4.821867244 | 5.55657028 |
| BMP6 | -0.735505783 | 0.600607426 | 0.001694721 | 0.143432071 | 0.750997417 | 1.486503199 |
| MT-TL1 | -0.735846889 | 0.600465437 | 0.02260033 | 0.271508961 | 4.377210998 | 5.113057887 |
| ABRACL | -0.740023876 | 0.598729443 | 0.004246506 | 0.170726769 | 2.265485166 | 3.005509042 |
| GATA3 | -0.743769039 | 0.597177188 | 0.000142203 | 0.072473812 | 1.229396157 | 1.973165196 |
| RPS27A | -0.745568055 | 0.596432982 | 0.04237725 | 0.343558805 | 5.174518066 | 5.920086121 |
| RPL32 | -0.749966861 | 0.594617216 | 0.031986963 | 0.307503181 | 5.759092273 | 6.509059134 |
| RPL6P27 | -0.751026388 | 0.594180684 | 0.000826522 | 0.121855199 | 1.973584846 | 2.724611234 |
| YBX1P10 | -0.752611 | 0.593528413 | 0.042430176 | 0.343558805 | 1.201285837 | 1.953896837 |
| RNF11 | -0.754766747 | 0.592642195 | 0.010255675 | 0.208438403 | 3.214750304 | 3.96951705 |
| AC116533.1 | -0.755200069 | 0.592464219 | 0.04151757 | 0.340321742 | 5.51678978 | 6.271989849 |
| IL2RB | -0.759428123 | 0.590730447 | 0.03306421 | 0.31015553 | 3.177021364 | 3.936449487 |
| GPR146 | -0.764517922 | 0.588650034 | 0.026947168 | 0.288794919 | 0.644480792 | 1.408998714 |
| S1PR1 | -0.769525294 | 0.586610462 | 0.011461146 | 0.218404293 | 2.136514547 | 2.906039841 |
| CABP5 | -0.774357026 | 0.58464913 | 0.025249513 | 0.281315602 | 0.901585443 | 1.675942469 |
| HSPA8 | -0.774958436 | 0.584405461 | 0.040369723 | 0.336368149 | 3.533576173 | 4.308534609 |
| RPS27AP16 | -0.775462391 | 0.584201355 | 0.022959931 | 0.273182337 | 2.600298156 | 3.375760547 |
| EIF2AK1 | -0.786552245 | 0.579727872 | 0.029402853 | 0.299226141 | 2.345096171 | 3.131648416 |
| RPL7P1 | -0.805616293 | 0.57211763 | 0.036217923 | 0.322226786 | 4.215961275 | 5.021577569 |
| RPL24P4 | -0.81133662 | 0.569853658 | 0.017241625 | 0.249927735 | 2.412754274 | 3.224090894 |
| TRAV8-4 | -0.8128769 | 0.569245584 | 0.021624424 | 0.2673433 | 1.08504759 | 1.897924489 |
| RPS7P1 | -0.814035408 | 0.568788654 | 0.004936246 | 0.176488433 | 3.330145098 | 4.144180505 |
| AC004086.1 | -0.826780997 | 0.563785783 | 0.001058079 | 0.124883735 | 3.660688307 | 4.487469304 |
| TENT5C | -0.826958089 | 0.563716582 | 0.038234065 | 0.329121424 | 1.219793363 | 2.046751452 |
| TRBV29-1 | -0.838336629 | 0.559288035 | 0.028675297 | 0.297514563 | 2.413493458 | 3.251830086 |
| MBNL3 | -0.848639176 | 0.555308284 | 0.018909953 | 0.259475892 | 1.269969853 | 2.118609029 |
| RPS20P14 | -0.852171211 | 0.55395043 | 0.044396526 | 0.350060123 | 2.322857832 | 3.175029043 |
| EEF1A1 | -0.8545193 | 0.55304957 | 0.011076537 | 0.214890011 | 7.240063609 | 8.094582909 |
| CTSW | -0.859691573 | 0.551070356 | 0.049610147 | 0.369300499 | 4.19739583 | 5.057087403 |
| LINC00989 | -0.873353203 | 0.545876614 | 0.00574663 | 0.18335663 | 1.442022518 | 2.315375721 |
| ST13 | -0.875384042 | 0.54510874 | 0.002415189 | 0.15111156 | 2.5515395 | 3.426923541 |
| RPSAP58 | -0.883558939 | 0.542028667 | 0.007072033 | 0.190773461 | 2.598241122 | 3.481800061 |
| TAL1 | -0.891198979 | 0.539165848 | 0.04271902 | 0.344773087 | 1.348246723 | 2.239445701 |
| PPM1A | -0.914577481 | 0.530499214 | 0.00291967 | 0.156159451 | 1.443610491 | 2.358187973 |
| YBX1P1 | -0.915340135 | 0.53021885 | 0.043108049 | 0.345718789 | 0.99991446 | 1.915254595 |
| RPL4 | -0.931121542 | 0.524450479 | 0.001942099 | 0.143432071 | 4.856095342 | 5.787216884 |
| RPS3A | -0.942892077 | 0.520189045 | 0.01255258 | 0.223934 | 5.225415743 | 6.16830782 |
| RPL5 | -0.947170814 | 0.518648557 | 0.005335321 | 0.179567556 | 4.169289815 | 5.116460629 |
| CAVIN2 | -0.949147142 | 0.517938554 | 0.027237114 | 0.289897629 | 2.570797263 | 3.519944405 |
| GTF3A | -0.952485717 | 0.516741366 | 0.000652759 | 0.109965644 | 1.687144524 | 2.639630241 |
| RPS6 | -0.961017797 | 0.513694383 | 0.001636346 | 0.143432071 | 5.541964028 | 6.502981825 |
| RPL12 | -0.974017986 | 0.509086253 | 0.005167039 | 0.178282889 | 6.579561061 | 7.553579047 |
| BTF3 | -0.976448529 | 0.508229305 | 0.021035061 | 0.266811776 | 5.101112134 | 6.077560663 |
| FUNDC2 | -0.979858748 | 0.50702938 | 0.015463711 | 0.239956533 | 1.408333748 | 2.388192496 |
| EEF1A1P5 | -0.991169871 | 0.503069674 | 0.007699492 | 0.195055265 | 4.95842884 | 5.949598711 |
| MMD | -1.029345183 | 0.489932471 | 0.007056732 | 0.190773461 | 1.863899964 | 2.893245148 |
| AP001324.1 | -1.0369218 | 0.487366232 | 0.014745371 | 0.237365813 | 4.258580506 | 5.295502307 |
| RPL7AP6 | -1.04272202 | 0.485410755 | 0.027244596 | 0.289897629 | 3.165974689 | 4.208696709 |
| LINC01578 | -1.047898647 | 0.483672143 | 0.004283749 | 0.170726769 | 1.86267056 | 2.910569207 |
| IGLV3-25 | -1.068825253 | 0.476707011 | 0.029781909 | 0.300929118 | 1.202799275 | 2.271624528 |
| PCGF5 | -1.073422615 | 0.475190331 | 0.032771953 | 0.308688012 | 2.875491976 | 3.94891459 |
| AC011979.1 | -1.088205129 | 0.470346173 | 0.00175008 | 0.143432071 | 1.012408302 | 2.100613431 |
| RPL21P75 | -1.089360702 | 0.469969585 | 0.004899573 | 0.176488433 | 2.827563633 | 3.916924335 |
| RPL21P16 | -1.094819443 | 0.468194716 | 0.007916369 | 0.195754223 | 4.843678843 | 5.938498287 |
| UBA52 | -1.102091547 | 0.465840653 | 0.023597415 | 0.276006413 | 7.950588726 | 9.052680274 |
| TRDC | -1.107220263 | 0.464187551 | 0.001469563 | 0.136271371 | 1.435997228 | 2.543217491 |
| SNHG5 | -1.12552186 | 0.4583362 | 0.022347706 | 0.271508961 | 1.589707527 | 2.715229388 |
| RPL41P1 | -1.13290447 | 0.455996776 | 0.018336964 | 0.257064018 | 6.365632961 | 7.498537431 |
| GSPT1 | -1.135076719 | 0.455310704 | 0.045826148 | 0.35628329 | 1.9325368 | 3.067613519 |
| LGALS3 | -1.146312133 | 0.451778609 | 0.045481395 | 0.355153841 | 4.020082546 | 5.166394679 |
| RPS12 | -1.165827188 | 0.445708634 | 0.004816002 | 0.175705137 | 9.278487037 | 10.44431422 |
| RUNDC3A | -1.18442187 | 0.440000824 | 0.049435924 | 0.369300499 | 2.244196319 | 3.428618189 |
| RGS10 | -1.204382979 | 0.4339549 | 0.019249563 | 0.261617729 | 4.700860448 | 5.905243428 |
| BAG1 | -1.261668133 | 0.417061449 | 0.030871291 | 0.304135878 | 4.35796239 | 5.619630523 |
| TPT1 | -1.293568133 | 0.407940844 | 0.002532176 | 0.15128476 | 5.461199808 | 6.754767941 |
| MXI1 | -1.307524654 | 0.404013483 | 0.006924794 | 0.188985286 | 1.851224712 | 3.158749366 |
| RPL21 | -1.318049894 | 0.401076712 | 0.002315389 | 0.14828481 | 6.35631798 | 7.674367874 |
| RPL7P9 | -1.327620527 | 0.398424832 | 0.007655467 | 0.195055265 | 3.982225216 | 5.309845743 |
| KLRB1 | -1.348472768 | 0.392707548 | 0.000174013 | 0.072473812 | 2.17595669 | 3.524429458 |
| IFIT1B | -1.465882192 | 0.362014103 | 0.006301313 | 0.186359769 | 0.870263519 | 2.336145711 |
| MYL4 | -1.472737817 | 0.360297909 | 0.045198395 | 0.354186718 | 5.042440278 | 6.515178095 |
| AC100810.1 | -1.489622984 | 0.356105597 | 0.017897173 | 0.253547252 | 2.857400945 | 4.347023929 |
| YBX1 | -1.496863459 | 0.354322882 | 0.007225944 | 0.191444492 | 6.389478055 | 7.886341514 |
| MAP3K7CL | -1.527021852 | 0.346992924 | 0.010610664 | 0.212260976 | 1.944692962 | 3.471714814 |
| OPTN | -1.582218911 | 0.33396784 | 0.012288366 | 0.2225944 | 2.208317635 | 3.790536546 |
| YBX3 | -1.613874822 | 0.326719661 | 0.026731258 | 0.288794919 | 3.104982687 | 4.718857509 |
| RN7SL2 | -1.63528594 | 0.3219066 | 0.033579966 | 0.311708921 | 5.885953361 | 7.521239301 |
| FBXO7 | -1.643853238 | 0.320000655 | 0.01566595 | 0.240016619 | 4.428094203 | 6.071947441 |
| STRADB | -1.64703096 | 0.319296588 | 0.04995129 | 0.369676686 | 2.934705438 | 4.581736398 |
| TUBB2A | -1.822712364 | 0.282688997 | 0.01800128 | 0.253547252 | 2.456154422 | 4.278866785 |
| C9orf78 | -1.930447941 | 0.262347702 | 0.000330835 | 0.083896316 | 2.820852777 | 4.751300718 |
| RPSAP15 | -1.957329827 | 0.257504611 | 0.005656319 | 0.183035808 | 0.816992187 | 2.774322014 |
| AC092490.1 | -2.218294426 | 0.214895261 | 0.002198162 | 0.147692828 | 5.059606397 | 7.277900823 |

NMDAR: The average expression value of genes in six samples of anti-NMDAR encephalitis. CON: The average expression value of genes in the six samples of the control group.
