# Supplementary material for: Immune inflammatory regulation in Anti-NMDAR encephalitis: insights from transcriptome analysis
Source: Front Neurol. 2025 May 9;16:1568274. doi: 10.3389/fneur.2025.1568274 (PMC12098042; doi:10.3389/fneur.2025.1568274)
Supplement: Supplementary Table 3 — 78 overlapping genes. [file Table_3.docx]

Supplementary Table 3 78 overlapping genes

| Gene_Name |
| --- |
| TPT1 |
| HSPA8 |
| S1PR1 |
| IL2RB |
| BMP6 |
| XCL2 |
| HSP90AB1 |
| NFATC2 |
| CD28 |
| CD247 |
| SH2D1A |
| CD40LG |
| PDCD1 |
| IL24 |
| KLRD1 |
| DCK |
| NCR1 |
| HMGB1 |
| FASLG |
| PLCG1 |
| TXK |
| CACYBP |
| ELAVL1 |
| MMP9 |
| RETN |
| SLPI |
| OPRL1 |
| NOD2 |
| S100A8 |
| LTB4R |
| GRN |
| S100A9 |
| HLA-A |
| S100A6 |
| ZYX |
| CD14 |
| CMTM2 |
| ADM |
| NLRX1 |
| SLC11A1 |
| FPR1 |
| S100A11 |
| LILRB3 |
| TYROBP |
| SEMA4A |
| HSPA1A |
| PLAUR |
| ITGB2 |
| OGFR |
| CSF3R |
| IL17RA |
| SEMA4B |
| RARA |
| IL4R |
| FGR |
| FCER1G |
| CMTM7 |
| HMOX1 |
| TNFRSF1A |
| RAC2 |
| NR1H2 |
| TNFRSF1B |
| PTPN6 |
| TYK2 |
| SH3BP2 |
| CXCR4 |
| PTK2B |
| TNFRSF12A |
| CMTM3 |
| PLXND1 |
| HCK |
| INSL3 |
| CSK |
| CSRP1 |
| RELA |
| LRSAM1 |
| IL2RG |
| TNFSF12 |
