# Supplementary material for: Immune inflammatory regulation in Anti-NMDAR encephalitis: insights from transcriptome analysis
Source: Front Neurol. 2025 May 9;16:1568274. doi: 10.3389/fneur.2025.1568274 (PMC12098042; doi:10.3389/fneur.2025.1568274)
Supplement: Supplementary Table 4 — NET-related genes among 899 differentially expressed genes. [file Table_4.docx]

Supplementary Table 4 NET-related genes among 899 Differentially expressed genes

| Gene_Name |
| --- |
| HMGB1 |
| MMP9 |
| PADI4 |
| DYSF |
| MGAM |
| FPR1 |
| ITGAM |
| BST1 |
| ITGB2 |
| CSF3R |
| CEACAM3 |
| LILRB2 |
| TECPR2 |
| SELPLG |
| RIPK3 |
